# Bone marrow microenvironment in autoimmune hemolytic anemia: from trephine biopsy to single cell RNA sequencing

**DOI:** 10.1038/s41392-025-02348-y

**Published:** 2025-08-25

**Authors:** Bruno Fattizzo, Matteo Claudio Da Vià, Francesca Lazzaroni, Alfredo Marchetti, Alessio Marella, Akihiro Maeda, Antonio Giovanni Solimando, Loredana Pettine, Francesco Passamonti, Niccolò Bolli, Wilma Barcellini

**Affiliations:** 1 Fondazione IRCCS Ca’ Granda Ospedale Maggiore Policlinico, Milan, Italy; 2 Department of Oncology and Haemato-Oncology, University of Milan, Milan, Italy; 3 Department of Precision and Regenerative Medicine and Ionian Area (DiMePRe-J), Unit of Internal Medicine “Guido Baccelli”, University of Bari “Aldo Moro” Medical School, Bari, Italy

**Keywords:** Haematological diseases, Immunological disorders

## Abstract

The role of bone marrow (BM) compensatory response in autoimmune hemolytic anemias (AIHAs) is emerging and inadequate reticulocytosis has been associated with more severe disease and adverse outcomes. However, few is known about the BM immunologic microenvironment composition in these diseases. Here we investigated BM features in a large cohort of 97 patients with autoimmune hemolytic anemia (AIHA) and observed a high prevalence of hypercellularity, dyserythropoiesis, reticulin fibrosis, and T-cell infiltration (65%, 29%, 76%, and 69% of patients, respectively). These findings were associated with inadequate bone marrow compensation, more severe anemia at onset, and need of multiple treatments. In a subset of warm type AIHA patients we investigated BM microenvironment by single-cell RNA sequencing. We found distinct immune cell profiles across disease stages (diagnosis, remission, relapse). In particular, upregulation of inflammatory response pathways was noted in CD8 + , CD4 + , and monocyte subsets during relapse compared to diagnosis and remission. Moreover, by single-cell TCR sequencing, we found small T cell clones at diagnosis that may either disappeared or expanded at remission. Disappearing clones exhibited a naive CD8+ phenotype and were more likely to respond to glucocorticoid treatment. Expanding clones showed upregulation of cytotoxic T cell markers and may play a role in the transition to a chronic/relapsing phase. Finally, cytokine gene expression differed across disease phases. At relapse, pro-inflammatory cytokines such as TNF-alpha, IL-1, and IL-6 were upregulated in CD4+ and CD8 + T cells, while TGF-beta was downregulated, potentially in an attempt to counteract the transition to chronic phase. This is the largest study evaluating BM histology and clinical characteristics, and the first evaluation of BM microenvironment by single-cell RNA sequencing in AIHA. We showed a complex scenario encompassing T-cell infiltration, clonality, and up/down-regulation of cytokine genes, associated with a more severe and relapsing disease.

## Introduction

Autoimmune hemolytic anemia (AIHA) is due to autoantibodies directed against antigens on the red blood cells.^1–6^ The disease is diagnosed in the presence of anemia of various degrees with hemolytic features (i.e., increased LDH, haptoglobin consumption, indirect hyperbilirubinemia, and increased reticulocyte counts) through the direct antiglobulin test (DAT), which is able to demonstrate the autoantibodies adherent on red blood cells surface.^1–6^ AIHAs are classified according to the DAT positivity and to the thermal range of the autoantibody into warm AIHA (wAIHA, DAT+ for IgG or IgG + C at low titer), cold AIHA (DAT+ for C and cold agglutinin titer >64 at 4 °C), mixed AIHA (DAT+ for both IgG and C at high titer), and atypical AIHA (DAT+ for IgA; warm IgM or DAT-negative). The different forms recognize a diverse pathogenesis of the hemolysis, in fact, warm reactive IgG opsonize the erythrocyte, are recognized by the Fc gamma receptor of spleen macrophages and lymphocytes and lead to antibody dependent cellular cytotoxicity (ADCC) and IgG-mediated extravascular hemolysis.^2^ On the other side, cold-reactive IgM are able to activate complement via the classical pathway leading to C3d deposition on red blood cells and subsequent complement mediated extravascular hemolysis in the liver (where C3 receptor is expressed). Notably, in case of infections, surgery traumas etc. complement cascade may be overactivated and cause intravascular hemolysis via the formation of the membrane attack complex (MAC) resulting in severe anemia and hemoglobinuria. Additionally, complement activation may occur even in about 30–50% of warm AIHA patients, particularly with some IgG idiotypes like IgG1 and 3, being associated with a more severe disease.^5,6^

Clinically, AIHAs are greatly heterogeneous, ranging from mild/compensated forms to life-threatening ones.^1–6^ The latter may require admission to the intensive care unit and aggressive treatment with steroids, transfusions, intravenous immunoglobulins, and plasmapheresis, and the mortality may be high.^2^ Along with autoantibody thermal amplitude, efficiency in activating complement, degree of mononuclear/phagocyte system activation, the efficacy of bone marrow (BM) compensatory response also plays an important role in determining disease severity.^7–9^ BM efficiently in compensating immune mediated hemolysis is generally mirrored by absolute reticulocyte counts, which may be however inadequate to the severity of anemia in about 20–30% of patients. A practical tool to assess the appropriateness of BM response is the bone marrow responsiveness index (BMRI), which is calculated as [patient’ absolute reticulocytes/mcL x (patient Hb g/dL / normal Hb g/dL]/1000”^7^ and reticulocytosis is considered inadequate for BMRI values lower than 121. Endogenous erythropoietin levels may also be evaluated and matched with Hb levels to assess adequacy.^8^

BM aspirate and trephine are not routinely performed in patients with primary AIHA, so the BM immunological microenvironment has not been systematically studied so far. Preliminary data showed that anemia severity is associated with inadequate reticulocytosis, possibly representing an autoimmune reaction against bone marrow erythroblasts.^10^ Consistently, the presence of BM hypercellularity, dyserythropoiesis, and fibrosis has been reported in about 1/3 of AIHA patients, with a proinflammatory cytokine signature, particularly in relapsed/refractory ones.^10^ These patients show similar features to bone marrow failure syndromes, may benefit of recombinant erythropoietin, and may eventually evolve into myelodysplastic forms.^10–15^

AIHAs are generally treated with immunosuppressive agents, particularly steroids and the anti-CD20 monoclonal antibody rituximab, with overall response rates exceeding 50% with differences among the various forms. In particular, wAIHA respond very well to frontline steroids in up to 70–80% of cases, and rituximab as second line induces about 70–80% of efficacy, long lasting in about a half of cases. In cold AIHAs, rituximab is advised as first-line with responses in about 50–60% of patients, mainly partial. The inhibition of the classical complement cascade by the anti-C1s monoclonal antibody sutimlimab may reduce hemolysis and improve anemia in more than 70% of patients with cold AIHA and such approach had been recently licensed for this condition. Additionally, other complement inhibitors and several new drugs targeting B-cells, plasma-cells, the monocyte-macrophage system, and the recycling of the pathogenic IgGs are under active investigation in both warm and cold forms.^1–3,5,6^

Despite such innovations, overall, about 1/3 of patients may be refractory to frontline treatment, and about 2/3 may relapse after an initial response thus becoming “chronic”. The physiopathology of the transition from the acute to the chronic/relapsing phase of AIHA is still unknown and likely reckons several mechanisms including the persistence of self-reactive B-cells, disturbances in T-cell subpopulations, and a peculiar cytokine imbalance. The investigations of the molecular mechanisms leading to disease refractoriness, relapse and transition to the chronic phase might inform the development of novel treatment approaches to overcome these unmet clinical needs.

In this study, we systematically evaluated BM features of a large cohort of AIHA patients, focusing on their association with clinical severity, response to therapy, and outcome. Furthermore, we pioneered the analysis of BM immunologic microenvironment by single cell RNA sequencing to evaluate different immune cells subpopulations and cytokine expression.

## Results

### Demographics and laboratory parameters

Ninety-seven patients have been included in the study. Median age was 59 years (range 10–89), and 40% of patients were elderly (>65 years). Median follow up from AIHA diagnosis was of 42 months, and AIHA type was equally distributed between warm (44%, 12 IgG + C) and cold forms (46%). 12% of patients had an associated condition: 9 lymphoproliferative syndromes, 2 myelodysplastic syndromes, and 1 thalassemia intermedia. Regarding hematologic parameters, 51% of cases presented with severe anemia (Hb <8 g/dL) and 19% with very severe anemia (Hb <6 g/dl). More than half of patients (56%) displayed inadequate reticulocytosis as depicted by a BMRI < 121, and 75% of patients had inadequate endogenous erythropoietin levels. During the follow up, 99% of patients received at least one treatment, 44% received 2 lines, and 31% 3 or more; therapies included steroids (*N* = 83), rituximab (*N* = 89), splenectomy (*N* = 3), and cytotoxic immunosuppressors (*N* = 25); 35 patients were also treated with recombinant erythropoietin due to inadequate bone marrow response (Supplementary table 1). Finally, 29% of patients experienced an infectious complication, 20% a thrombosis, and 6% an acute renal failure in keeping with available literature.^16–18^

### Bone marrow characterization of AIHA patients

Table 1 shows clinical and laboratory parameters divided according to bone marrow features. Hypercellularity, present in the majority of patients (65%), was associated with higher hemolytic features (greater LDH, unconjugated bilirubin levels and compensatory reticulocytosis in hypercellular versus normo/hypocellular patients *p* = 0.05, *p* = 0.01, and *p* = 0.05, respectively). Reticulin fibrosis (MF-1) was observed in 29% of cases, and dyserythropoiesis in 76%, associated with older age (*p* = 0.009) and wAIHA subtype (*p* = 0.04). As shown in Table 2, a lymphoid infiltrate was documented in 85% of patients, was ≥10% in 20% of cases, and more pronounced in mixed AIHA subjects (*p* = 0.005). The infiltrate showed a T-cell phenotype in about 50% of cases, a mixed T/B phenotype in 40%, and B-cell one in 10%, mainly cAIHA patients (*p* = 0.05). B-cell lymphoid infiltrate was clonal in 15 patients only (18%), mainly with primary cAIHA (*N* = 10, cold agglutinin associated lymphoproliferative syndromes, *p* = 0.03), or secondary cAIHA (3 chronic lymphocytic leukemia, and 2 IgG MGUS). T-cell infiltrate was associated with lower Hb levels and with inadequate endogenous erythropoietin levels (*p* = 0.003). Cytogenetics aberrations were found in 8% of subjects, including chromosome Y deletion (3%), 1 inversion, 20q deletion, trisomy 8, and 1 translocation (X,20)(q13;q13). Other variables including blood counts, serum Ig levels, and presence of splenomegaly were comparable among groups with different bone marrow features. Regarding treatment, T-cell infiltrate was associated with higher transfusion need at onset (45 versus 32% in those with B- and mixed B/T infiltrate) and with the occurrence of 2 or more relapses. B-cell infiltrate was associated with complete response to rituximab (75 versus 40% in those with mixed T/B and T-cell infiltrate). Contrarily, lower response rates to rituximab were associated with hypercellularity and dyserythropoiesis (Supplementary Tables 2 and 3).

**Table 1:** Clinical and laboratory parameters of autoimmune hemolytic anemia (AIHA) patients according to bone marrow features

	Normo/ hypocellularity (*N* = 34)	Hypercellularity (*N* = 63)	Diserythropoiesis (*N* = 74)	No diserythropoiesis (*N* = 23)	MF - 0 (*N* = 69)	MF- 1 (N = 28)
Age, years*	61 (21–82)	59 (10–89)	64 (20–89)^c^	55 (10–71)	60 (21–89)	58 (10–85)
Warm AIHA n (%)	13 (38)	30 (48)	37 (50)^c^	6 (26)	41 (60)	15 (53)
Cold AIHA^a^ n (%)	18 (53)	26 (41)	29 (39)	15 (65)	23 (33)	8 (29)
Mixed AIHA n (%)	1 (3)	2 (3)	2 (3)	1 (4)	1 (1)	2 (7)
Atypical AIHA^b^ n (%)	2 (6)	5 (8)	6 (8)	1 (4)	4 (6)	3 (11)
Hb (g/dl)	8.3 (4.0–11.6)	7.5 (3.0–11.5)^c^	7.7 (3.8–11.5)	8.2 (3.0–11.6)	7.9 (3.0–11.6)	8.1 (4.0–11.5)
LDH (U/L)	325 (155–1199)	506 (182–2617)^c^	451 (155–2617)	503 (194–1153)	460 (155–1500)	478 (194–2617)
Unconjugated bilirubin (mg/dl)	1.3 (0.4–3.4)	2.2 (0.3–9.6)^c^	1.6 (0.3–9.6)	2.1 (0.9–5.8)	1.7 (0.3–9.6)	2.5 (0.7–7.5)
Ret (x10^a^9/L)	156 (4.3–660)	207.5 (1.5–623)^c^	201 (4.3–623)	161 (86–660)	178 (4.3–660)	198.5 (239-616)
BMRI**	74.5 (0.0–325.3)	91.7 (0.0–426.8)	98 (4–427)	96 (23–325)	104.1 (3.5–325.3)	90.9 (12.4–426.8)
Inadequate reticulocytosis*** n (%)	13/20 (65)	23/44 (52)	28/51 (55)	8/13 (62)	23/44 (52)	13/20 (65)
EPO (U/L)	38.4 (16.0–147.0)	65.0 (10.0–843)	57.5 (10–843)	51.2 (17.2–147.0)	55.3 (10.0–843)	51.2 (16.1–208.0)
Inadequate EPO n (%)	17/20 (85)	30/43 (70)	37/50 (74)	10/13 (77)	34/46 (74)	13/17 (76)

*MF0* without marrow fibrosis, *MF1* reticulin marrow fibrosis (grade 1 according to WHO), *AIHA* autoimmune hemolytic anemia, *Hb* hemoglobin, *LDH* Lactate dehydrogenase, *Hapto* haptoglobin, *Ret* reticulocytes, *EPO* endogenous erythropoietin, *WBC* white blood cells, *PLT* platelets

* Values are expressed as median (range). unless otherwise specified

**BMRI bone marrow responsiveness index = [absolute reticulocyte count x (patient’s Hb/normal Hb)]/1000

***BMRI < 121

^a^5 patients had a condition associated with their cAIHA: 3 had associated chronic lymphocytic leukemia and 2 had a IgG monoclonal gammopathy of unknown significance

^b^6 patients had DAT negative AIHA, 1 had IgA positive AIHA

^c^p ≤ 0.05

**Table 2:** Clinical and laboratory parameters of autoimmune hemolytic anemia (AIHA) patients according to bone marrow lymphoid infiltrate

	B-cells (*N* = 8)	T-cells (*N* = 40)	Mixed (*N* = 34)	No lymphoid infiltrate (*N* = 15)
Age, years*	58 (45–85)	60 (20–84)	59 (10–89)	59 (33–83)
Warm AIHA n (%)	1 (13)	21 (53)	15 (44)	6 (40)
Cold AIHA n (%)^a^	7 (88)	16 (40)	16 (47)	5 (33)
Mixed AIHA n (%)	0 (0)	0 (0)	2 (6)	1 (7)
Atypical AIHA n (%)^b^	0 (0)	3 (8)	1 (3)	3 (20)
Hb (g/dl)	8.2 (5.2–11.6)	7.3 (3.8–11.4)^c^	8.2 (4.0–11.5)	8.3 (3.0–9.8)
LDH (U/L)	513 (320–981)	509 (155–1763)	382 (195–2617)	445 (172–1500)
Unconjugated bilirubin (mg/dl)	2.1 (1.1–5.7)	2.1 (0.3–8.7)	1.6 (0.7–9.6)	1.7 (0.8–3.0)
Depleted hapto n (%)	5/5 (100)	24/24 (100)	25/29 (86)	6/7 (86)
Ret (x10^a^9/L)	222.5 (196–660)	245 (4.3–623)	161 (27-549)	171 (23.9-601)
BMRI**	148 (78–325)	107 (4–427)	90 (17–265)	97 (12–305)
Inadequate reticulocytosis*** n (%)	1/4 (25)	12/25 (48)	18/27 (67)	5/8 (63)
EPO (U/L)	65 (30–208)	51.2 (16–843)	61 (10–226)	55.3 (17.6–170)
Inadequate EPO n (%)	5/7 (71)	23/25 (92)^c^	11/21 (52)	8/10 (80)
WBC (x10^a^9/L)	5.7 (4.4–12.9)	7.2 (1.7–19.9)	5.5 (2.7–26.0)	5.3 (3.9–14.8)
PLT (x10^a^9/L)	292 (123–357)	249 (110–889)	242 (61–414)	207 (1–414)
IgG (mg/dl)	625 (131–1623)	863 (312–2758)	921 (217–1719)	930 (719–1030)
IgA (mg/dl)	99 (40–393)	195 (43–433)	150 (14–351)	140 (79–232)
IgM (mg/dl)	387 (52–6640)	85 (17–448)	130 (32–1867)	84 (19–136)
MGUS n (%)	3/8 (38)	3/40 (8)	4/34 (12)	0/15 (0)
Splenomegaly n (%)	3/8 (38)	15/40 (38)	15/34 (44)	7/15 (47)

*Mixed* B-cells + T-cells, *AIHA* autoimmune hemolytic anemia, *Hb* hemoglobin, *LDH* Lactate dehydrogenase, *Hapto* haptoglobin, *Ret* reticulocytes, *EPO* endogenous erythropoietin, *WBC* white blood cells, *PLT* platelets, *MC* monoclonal component, *aPL* antiphospholipid antibodies

* Values are expressed as median (range). unless otherwise specified

**BMRI bone marrow responsiveness index = [absolute reticulocyte count x (patient’s Hb/normal Hb)]/1000

^a^5 patients had a condition associated with their cAIHA: 3 had associated chronic lymphocytic leukemia and 2 had a IgG monoclonal gammopathy of unknown significance

***BMRI < 121

^b^6 patients had DAT negative AIHA, 1 had IgA positive AIHA

^c^p ≤ 0.05

### Single-cell RNA sequencing characterization of wAIHA patients

To better characterize bone marrow microenvironment composition and its impact on disease onset and therapy response we evaluated 9 wAIHA patients at single cell level. Table 3 shows clinical and hematological features of the patients included in the scRNAseq analysis divided according to the disease phase (diagnosis, remission, and relapse). As expected, anemia and alteration of hemolytic markers were present at diagnosis and relapse, and BM features were consistent with the overall wAIHA population. Regarding treatment, patients tested at diagnosis were on steroids since 2–5 days, those in remission were off treatment, and those evaluated at relapse had all received steroids and rituximab, and one danazol.

**Table 3:** Clinical and laboratory features of warm autoimmune hemolytic anemia patients evaluated by single-cell RNA sequencing at the time of sampling

	Sex	Age	Hb, g/dL	LDH, U/L	Total bilirubin mg/dL	Therapy	diserythropoiesis	Lymphoid infiltrate (% - type))	Fibrosis
Diagnosis									
Patient 1	F	51	4	1200	6.00	Steroid	Yes	10- T	MF-0/1
Patient 2	M	75	8.5	401	1.52	Steroid	Yes	5 T	MF-0
Patient 3	M	77	8.8	488	1.66	Steroid	No	10 T	MF-0
Patient 4	M	85	3	1251	10.16	Steroid	No	10 T	MF -0
Patient 5	M	85	6	981	6.60	Steroid	Yes	10 B	MF-1
Remission									
Patient 1	F	51	15.9	280	3.40	//	Yes	15 T	MF-1
Patient 2	M	75	13.5	197	0.52	//	Yes	14 T	MF-0
Patient 3	M	77	12.4	342	0.67	//	No	12 T	MF-0
Relapse									
Patient 6	M	77	6.4	1207	8.20	Steroid, rituximab	No	5 T	MF-0/1
Patient 7	F	68	7.6	440	1.50	Steroid, rituximab, danazol	Yes	15 T	MF-1
Patient 8	F	28	8.1	230	1.80	Steroid, rituximab	Yes	10 T	MF-0
Patient 9	F	41	6.8	1270	7.60	Steroid, rituximab	Yes	5 T	MF-0

**Fig. 1:**
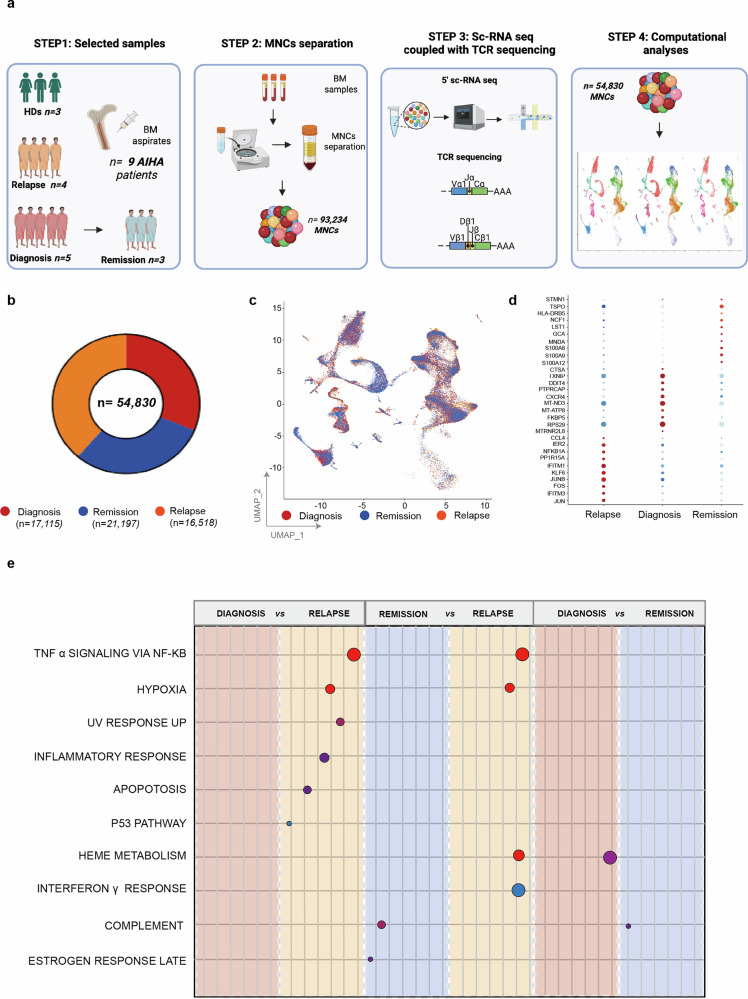
Single-cell RNA sequencing landscape of AIHA patients. **a** Schematic workflow for scRNA-seq of AIHA and healthy donors samples. The panel was created by Biorender.com. **b** Donut charts representing the distribution of cells in AIHA patients according to clinical stratification (Red: diagnosis; Orange: relapses/remission; blue: remission). Numerical values are reported in the legend for category. **c** Uniform Manifold Approximation and Projection (UMAP) representation of cells (*N* = 54,830) from the 9 AIHA single-cell RNA-seq data. Clusters of cells are colored by clinical stratification. Color legend as in (**b**). **d** Dot plot displaying the top ten marker genes that distinguish the clinical stratification. The X-axis lists the clinical category, while the Y lists gene names. Circle size corresponds to the number of cells in the category expressing the gene of interest, while shade correlates with the level of expression. **e** Cartoon depicting the deregulated expression profiles of AIHA patients, obtained from 1:1 DE analyses by Wilcoxon test. Color code as in Fig. 1b. Original plot results represented in Figure Supplementary Fig. 3

Figure 1a summarizes the workflow of scRNA-seq processing of 12 samples from 9 patients (5 patients sampled at diagnosis -3 of whom re-sampled at remission 6 months later- and 4 unrelated relapsed patients) and 3 healthy donors (Fig. 1a). After filtering cells using standard quality controls (Supplementary Fig. 1a–d), a total of *n* = 54,830 MNCs (*n* = 17,115 from samples at diagnosis, *n* = 16,518 at relapse and *n* = 21,197 at remission) were used for further analysis (Fig. 1b). By Uniform Manifold Approximation and Projection (UMAP) analysis and visualization of scRNA-seq data (Fig. 1c), MNCs cells from different samples and clinical stages clustered together (UMAP plots by clusters and by patients are reported in Supplementary Fig. 2a, b). Figure 1d summarizes the differential gene expression (DGE) analysis according to the different clinical stages: cells from relapsed patients showed an upregulation of genes related to inflammatory response (*i.e., IFITM1, IFITM3, NFKB1A* and *KLF6*) and immune response (*i.e., JUN, JUNB*) as compared to diagnosis and remission. Conversely, at diagnosis, an up-regulation of genes involved in cell motility/chemotaxis (such as *CXCR4*), in T cell activation (i.e., *PTPRCAP*) and interferon production (i.e., *FKBP5*) was noted. At remission, genes involved in T cell inhibition (i.e., *LST1 and MNDA*), T cell and myeloid lineage maturation, and S100s (*S100A8/9/12*) genes were upregulated. Accordingly, by gene set enrichment analysis (GSEA) at the level of “hallmark” gene sets (from the Human Molecular Signatures Database - MSigDB) (Fig. 1e, Supplementary Fig. 3a, b) an enrichment of pathways related to inflammatory response, including “TNFα signaling via NFκB”, “Inflammatory response”, “UV response”, “Hypoxia”, “Apoptosis” and “Interferon γ”, was found in relapsed patients compared with diagnosis and remission. Heme-methabolism and complement pathways were respectively enriched at diagnosis and remission (Fig. 1e, Supplementary Fig. 3c). In order to better assess the inflammatory status across the pathological disease status we performed a direct comparison with healthy donor (HD) derived BMs. Of note, the comparison of wAIHA patients with healthy donors showed a large overlap of BM environment composition except for a reduced B-cell population and an upregulation of interferon genes in the pathological setting (Supplementary Fig. 4a–e). Moreover, as reported in the GSEA, interferon and T cell immunity related pathways were strongly enriched in the disease setting in comparison to HDs Supplementary Fig. 5). These data suggest a strong persistent proinflammatory phenotype of the transcriptional landscape of relapsed wAIHA patients as compared to the diagnosis, remission and HDs.

### The bone marrow niche associated T cells, monocytes and macrophages landscape

**Fig. 2:**
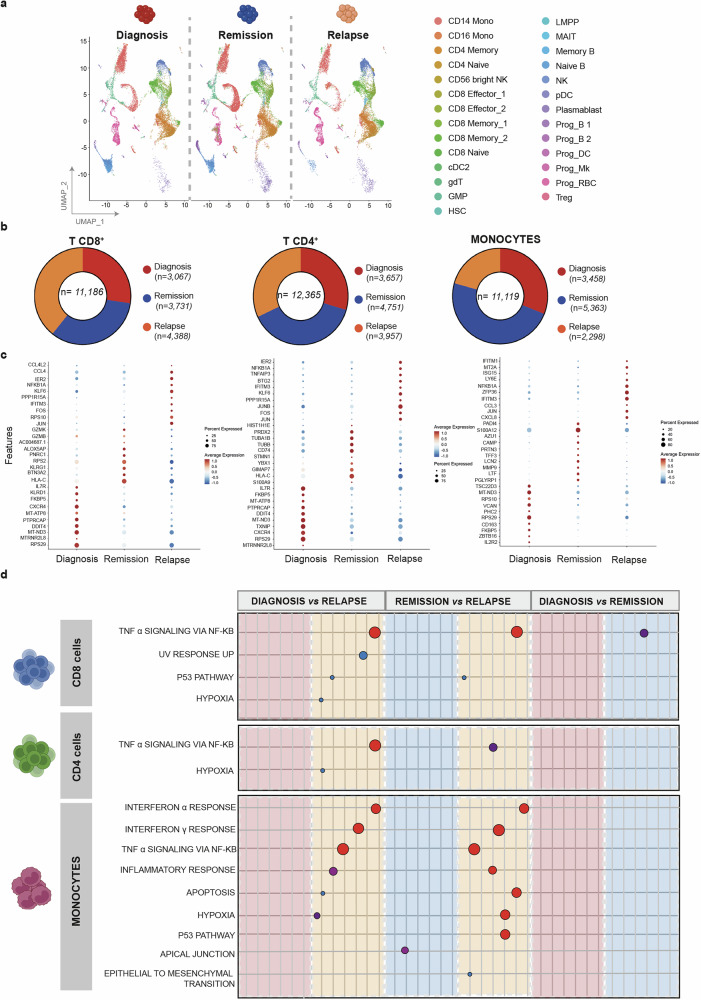
The bone marrow-associated cell repertoire of AIHA patients. **a** Single-cell uniform manifold approximation and projection (UMAP) plot of the AIHA patients. Cells are color-coded by computationally determined cell clusters. **b** Donut charts representing the distribution of immune cells (T CD8 + , T CD4+ and monocytes) in AIHA patients according to clinical stratification (Red: diagnosis; Orange: relapses/remission; blue: remission). Numerical values are reported in the legend for category. **c** Dot plot displaying the top ten marker genes that distinguish the clinical stratification, across immune cell repertoire (T CD8 + , T CD 4 + and monocytes). The X-axis lists the clinical category, while the Y lists gene names. Circle size corresponds to the number of cells in the category expressing the gene of interest, while shade correlates with the level of expression. **d** Cartoon depicting the deregulated expression profiles of AIHA patients, obtained from 1:1 DE analyses by Wilcoxon test, across immune cell repertoire (T CD8 + , T CD 4 + and monocytes). Color code as in Fig. 1b. Original plot results represented in Figure Supplementary Fig. 5

By comparing MNCs subsets among clinical stages (Fig. 2a), a decrease of CD14+ monocytes and of naïve and memory B cells was observed in relapsed patients as compared to diagnosis/remission. By re-annotating cells through ProjecTILs atlas,^19^ a reference cell atlas able to granularly annotate T cell and monocytes subpopulation, a total of 34,670 cells were characterized (11,186 CD8 T cells, 12, 365 CD4 T cells, and 11,119 monocytes) (Fig. 2b, Supplementary Fig. 6a–c), with similar cell abundance across disease phases. At remission, a trend for higher levels of CD8 memory T cells and CD16+ monocytes were observed versus diagnosis and relapse (CD16+ monocytes only). Interestingly, healthy donors showed a similar abundance of CD8 T cell memory as that observed in wAIHA patients at remission. Moreover, HD showed lower levels of CD8-naïve T cells, CD8 TEMRA T cells, and CD14+ monocytes but higher abundance of thrombospodin-expressor monocytes versus wAIHA patients (Supplementary Fig. 6a–c). Despite similar relative abundances, CD8, CD4 and monocytes differed significantly by DGE analysis (Fig. 2c, d**;** Supplementary Fig. 7): at relapse, an upregulation of inflammatory pathways (TNF-alpha signaling for all the three cell types and IFN pathways for monocytes only) was noted compared with diagnosis and remission. We further analyzed the CD169^pos^ macrophages which represented a low proportion of microenvironmental cells and showed a similar abundance across disease stages. Analysis of marker genes showed that disease stages differed in the expression of pro-inflammatory, autophagy modulators, anti-apoptotic signaling and NOTCH pathway activator genes (Supplementary Fig. 8). Interestingly, a different distribution of MHC class II antigens, mainly enriched at the remission status, was noted (Supplementary Fig. 7a). Additionally, GSEA analysis showed an upregulation of TNF-alpha signaling, as well as mTOR, hypoxia and apoptosis pathways significantly enriched in the relapse setting. (Supplementary Fig. 8b–d). These data corroborate a high chronic inflammatory state at relapse through activation of IFN related pathways and T cell skewed immunity, suggesting their role in the functional polarization and activation of BM resident macrophages. Finally, the analysis of FCGR1A, 2 A, 2B, 3 A and 3B genes did not show differential expression in monocytes at various disease stages (data not shown).

### T cell population specific TCR between disease stages show differential T cell clonal expansion

**Fig. 3:**
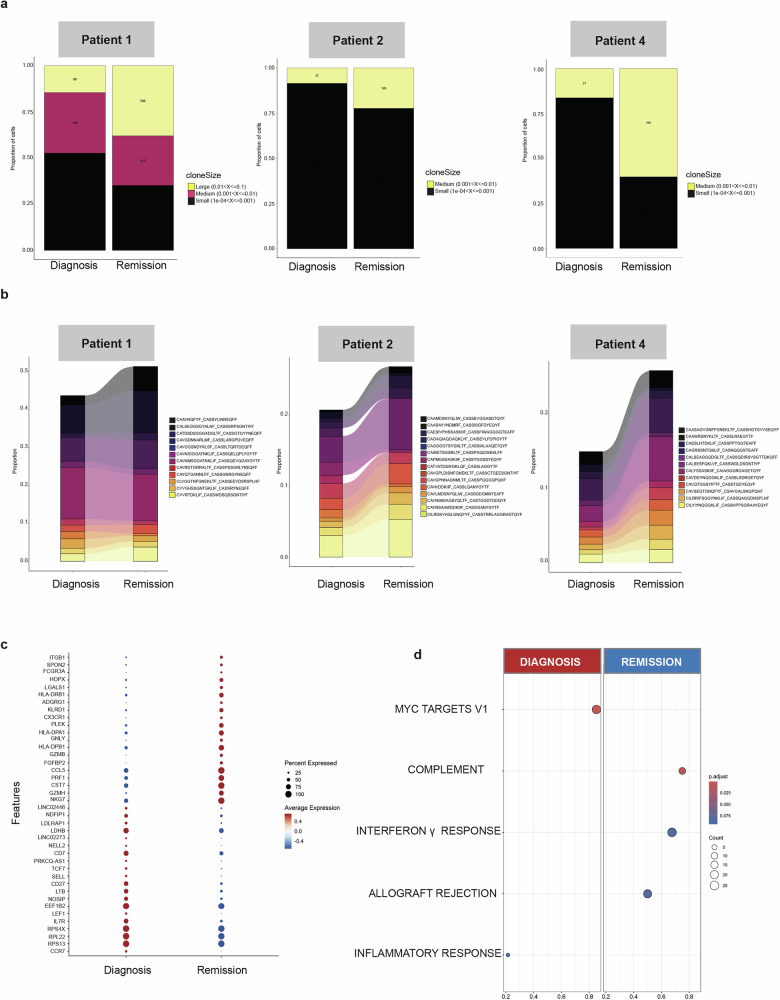
Transcriptional phenotype of CD8+ cell repertoire in bone marrow aspirates of AIHA patients. **a** Distribution of the CD8 T clone size among small, medium and large cell clones in bone marrow aspirates from three matched patients at diagnosis and remission. Colors represent group size from small to large size. **b** Alluvial plots showing the distribution of T cell clones dynamics from diagnosis to remission samples, according to the T cell clones. **c** Dot plot displaying the top ten marker genes of CD8 + T cells clones that distinguish the diagnosis and remission. The X-axis lists the clinical category, while the Y lists gene names. Circle size corresponds to the number of cells in the category expressing the gene of interest, while shade correlates with the level of expression. **d** Dot plots showing the Wilcoxon enriched hallmarks of CD8 + T cells clones of AIHA patients at diagnosis and remission. Gene-ratio on the y-axis, counts as dot size and color represent *padj* values in z-score

By single cell TCR analysis on CD8 and CD4 T cells from the 3 patients with serial evaluations at diagnosis and remission (patient 1, 2 and 4) we observed features of clonality. In detail, patient 1 showed large size clones (i.e., 1-10% of T-cell cellularity) at diagnosis; differently, patient 2 and 4 only displayed smaller clones. Overall, clone dynamics between diagnosis and remission showed a significant decrease of small clones (with some clones disappearing between the two time points) and expansion of medium and large ones in each subject (Fig. 3a, b). Different types of clonal dynamics could be observed: clones present only at diagnosis (namely singlets) and clones persisting or expanding within diagnosis and remission (dual expanded ones) (supplementary Fig. 9). By subsetting such clones, singlet ones showed mainly a naive CD8 + T cell phenotype and a general upregulation of genes related to cell metabolism (i.e., *LDHB, LDLRAP1, NOSIP*), Th17 subset and immune system development (i.e., *PRKCQ-AS1, NDFIP1, NELL2*) (Fig. 3c). Conversely, the dual expanded clones showed an upregulation of cytotoxic T cell markers (i.e., *PRF1, GNLY, CX3CR1, FCGR3A*), activator granzymes (*GZMB/H*), class II major histocompatibility complex genes (*HLA-DPB1, HLA-DPA1, HLA-DRB1*) and cell adhesion molecules and chemokines (i.e., *SPON2, CCL5, ITGB1*) (Fig. 3c). Of note, by GSEA, inflammatory and MYC related pathways were upregulated in singlets, while T cell specific pathways (allograft rejection and IFN-gamma) resulted upregulated in dual-expanded clones (Fig. 3d). Interestingly, the tracking of T cell populations through their specific TCR showed a differential clonal expansion between the two timepoints, suggesting huge differential susceptibilities to induction steroid therapy in peculiar cell subsets.

### Cytokine related signatures at single cell level show differential signaling pathways among disease stages

The gene expression profile of 42 cytokines was evaluated in different cell subpopulations, namely CD8+ effectors, CD8+ memory, CD4+ naive, CD4+ memory T cells, and CD14+ monocytes, and compared across disease phases (Table 4). By comparing remission versus diagnosis, TGF-beta and several TGF-beta superfamily cytokine genes (*BMP2, EGF, GDF11*) were up-regulated in CD8+ effector T cells; a similar profile was found in CD8+ memory T cells that also displayed down-regulated *IFN*, Th1 cytokine genes (*IL-2, IL-12, IL-15*), and the anti-inflammatory cytokine gene *IL-22*. No clear trends were observed in CD4+ naive and memory T cells, as well as in CD14+ monocytes. Regarding the comparison between relapse and diagnosis, TNF-alpha, TGF-beta and TGF-superfamily cytokine genes (*Activin A, BMP2 and 4*), as well as several growth factor genes (*G-CSF, GM-CSF, MN-CSF*), were up-regulated in CD8+ effectors, and to a lesser extent in CD8+ memory T cells.

**Table 4:** List of cytokines pathways up- and down-regulated in different immune cells (CD8 + , CD4 + T cells, CD14+ monocytes) across different timepoints (remission vs diagnosis and relapse vs diagnosis)

	Remission vs Diagnosis	Relapse vs Diagnosis
CD8+ Effector T cells	**Up-regulated**: BMP2, EGF, GDF11, TGFB3, NO **Down-regulated**: IL3	**Up-regulated**: Activin-A, BMP2, CD40L, CXCL12, GCSF, GMCSF, IL10, MCSF, TNFA, TGFBP3, NO **Down-regulated**: IL21
CD8+ Memory T cells	**Up-regulated:** BMP2, GCSF, MCSF, TGFB3 **Down-regulated**: IFNG, IL12, IL15, IL2, IL21, IL22, IL3, OSM, VEGFA	**Up-regulated**: BMP2, BMP4, GCSF, IL10, MCSF, NO **Down-regulated**: IL12, IL1A, IL21, IL36, VEGFA
CD4+ Naive T cells	**Up-regulated:** IL21, IL22, IL3 **Down-regulated:** EGF	**Up-regulated**: BDNF, BMP4, CD40L, GCSF, IL17A, IL1A, IL1B, IL27, IL6, LIF, MCSF, TGFB3, TNFA, NO, TWEAK **Down-regulated**: GMCSF, IL15, IL4
CD4+ Memory T cells	**Up-regulated**: GCSF	**Up-regulated**: BDNF, BMP4, CD40L, GCSF, IL17A, IL1A, IL1B, IL27, IL6, LIF, MCSF, TGFB3, TNFA, NO, TWEAK **Down-regulated**: GMCSF, IL15, IL4
CD14+ Monocytes	**Up-regulated:** IL2, VEGFA **Down-regulated**: GDF11, TGFB3	**Up-regulated**: EGF, FGF2, GMCSF, IL12, IL15, IL2, IL21, IL22, IL3, IL36, LTA, OSM, VEGFA **Down-regulated:** GDF11, IL13, IL27, LIF, TGFB1, TGFB3, TWEAK

Only statistically significant differences with an adjusted *p*-value < 0.001, as determined by CytoSig analysis, are reported

Interestingly, expression of TGF-beta and TGF-beta superfamily genes (*EGF, GDF11*) was down-regulated in both CD4+ naive T cells and CD14+ monocytes. At variance, TNF-alpha and its superfamily genes *TWEAK*, *IL-1, IL-6, IL-17, IL-27* were up-regulated in CD4+ memory T cells. Detailed trends of cytokines are shown in Supplementary Figs. 10 and 11.

## Discussion

In this study we firstly investigated BM features in a large cohort of patients with AIHA, and showed a high prevalence of hypercellularity, dyserythropoiesis, and reticulin fibrosis (65%, 29% and 76% of patients, respectively) as well as of T-cell infiltrate (69% of subjects), being associated with inadequate bone marrow compensation (i.e., inappropriate reticulocyte response and endogenous erythropoietin levels), more severe anemia at onset, and higher number of therapy lines. Notably, the prevalence of inadequate reticulocytosis is increasingly recognized in AIHA patients and was high in this patient population, justifying the severity of anemia. By applying the bone marrow reticulocyte index the frequency of inappropriate bone marrow compensation increased from 18 to 50% in two large retrospective studies.^7,8^ The association of impaired BM compensation and features of hypercellularity may imply an “ineffective erythropoiesis” associated with an immune attack against BM precursors, a pro-inflammatory BM milieu, and inadequate levels of endogenous erythropoietin, as already described.^10,20,21^ These findings depict a complex scenario where T-cells are not simply fostering humoral anti-erythrocyte autoimmunity but exert a cytotoxic effect in a proinflammatory/proapoptotic BM microenvironment. In fact, single cell RNA sequencing highlighted distinct immune cell features in different disease stages, namely diagnosis, remission, and relapse. In particular, despite a similar “quantitative” composition of T-cell subpopulations and monocytes across disease’s phases, significant “qualitative” functional differences emerged by differentially expressed genes (DEGs) and GSEA analysis. This is particularly evident at relapse compared to diagnosis and remission, where an upregulation of pathways related to inflammatory response was noted both in total MNCs and in CD8 + , CD4+ and monocytes subsets. In particular, a functional skew towards T cell immune activation was noted as compared to healthy donors, further highlighting a likely pathogenetic role of this cell population. Interestingly in our study, for the first time, small T cell clones were dissected by single-cell TCR analysis. Clones present at diagnosis may either disappear or expand at remission. Those disappearing at remission showed mainly a naive CD8 + T cell phenotype, a general upregulation of genes related to cell metabolism, Th17 subset and immune system development, and might be more sensitive to glucocorticoid treatment. Conversely, clones persisting/expanding at remission showed an upregulation of cytotoxic T cell markers, activatory granzymes, class II major histocompatibility complex genes, cell adhesion molecules, and chemokines, and might play a role in the disease onset and in transition to the chronic phase. Moreover, the significantly enrichment of MYC related pathways in the T cell population at diagnosis may imply an activation of T cell differentiation pathways from naïve T cells, suggesting a direct involvement of the T cell populations in the pathogenesis and maintenance of the disease, as reported for systemic lupus erythematosus.^22,23^

Several cytokines and chemokines have been shown to be deregulated in wAIHA.^24^ In our study, the different expression of cytokine genes across AIHA disease phases further support the contribution of T-cells and monocytes to the pro-inflammatory bone marrow milieu.^21,25,26^ At relapse, TNF-alpha and its superfamily genes, IL-1, IL-6, IL-17, IL-27 were upregulated in both CD4+ memory and in CD8+ effectors and memory cells. Consistently, the regulatory cytokine TGF-beta and TGF-superfamily genes were downregulated in CD4+ naive cells and CD14+ monocytes; while they were upregulated in CD8+ effectors and memory cells, as a possible mechanism to counteract the transition to the chronic phase. In keeping with the anti-inflammatory activity of this cytokine, the remission phase was characterized by an upregulation of TGF-beta and several TGF-beta superfamily cytokines in CD8+ effector and memory cells. Moreover, remission was marked by a downregulation of IFN-gamma, Th1 cytokines (IL-2, IL-12, IL-15), and the anti-inflammatory cytokine IL-22 genes, likely indicating a general “shut down” of the autoimmune response.^21,24,27^

Our study carries some limitations, including the small number of cases evaluated by scRNAseq either at diagnosis or during different disease phases. However, BM is not routinely performed in wAIHA patients, and BM features of this subgroup were consistent with the whole wAIHA population, where histopathological features were related with clinical outcomes. Cold AIHA category is overrepresented in this series as compared to the reported frequency of 20–30%. This might be attributed to our center being a referral for AIHA. However, scRNAseq analysis was intentionally performed in wAIHA cases only, since cold agglutinin disease is a known lymphoproliferative disorder, recently included in the WHO 2022, that might bias the immunological analysis. For the same reason secondary cold AIHA were also excluded. Regarding the possible effect of treatment on BM features, in the histopathological part of the study patients with cold AIHA underwent bone marrow evaluation at diagnosis before rituximab treatment. Differently, warm type AIHA were evaluated at relapse after the first course of steroids, so that a possible effect of steroids cannot be excluded. The same applies to scRNAseq results and is in keeping with the acute/unpredictable nature of wAIHA presentation requiring prompt treatment. Finally, it may also be argued that we mainly focused on T-cells and monocytes. As a matter of fact, the initial evaluation of all BM cells revealed that T-cells and monocytes were the most abundant and therefore the analysis focused on these subpopulations. Additionally, the histopathological part of the study had provided hints of an association between T-cell infiltrate and clinical features of the disease.

In conclusion, this first evaluation of the BM microenvironment in AIHA revealed a complex scenario marked by a T-cell infiltration, clonality, and up- or down-regulation of several cytokine genes being associated with a more severe and relapsing disease and with a possible chronic evolution.

## Material and methods

Patients with AIHA diagnosis undergoing BM evaluation at Fondazione IRCCS Ca’ Granda Ospedale Maggiore Policlinico of Milan from June 1987 to November 2021 were included. AIHA diagnosis was established in agreement with current recommendations^2^ and patients were classified according to the positivity of the direct antiglobulin test (DAT) and to the thermal range of the autoantibody into warm AIHA (wAIHA, DAT+ for IgG or IgG + C at low titer), cold AIHA (DAT+ for C and cold agglutinin titer ≥64 at 4 °C), mixed AIHA (DAT+ for both IgG and C at high titer), and atypical AIHA (DAT+ for IgA; warm IgM or DAT-negative). Clinical and hematologic parameters were retrospectively collected. Bone marrow responsiveness index (BMRI) was calculated as [patient’ absolute reticulocytes ×(patient Hb/normal Hb)]/1000 and reticulocytosis was deemed inadequate for BMRI < 121. Endogenous erythropoietin was also evaluated and matched with Hb levels to assess adequacy.^8,12^ The following therapy lines were considered: steroids, intravenous immunoglobulin, cytotoxic immunosuppressants, rituximab, splenectomy, recombinant erythropoietin, eculizumab, and bortezomib. Response to therapy was considered complete (CR) for Hb ≥ 12 g/dL and partial (PR) for Hb ≥ 10 g/dL or increase of at least 2 g/dL from baseline. Complications were collected and graded according to the Common Terminology Criteria for Adverse Events (CTCAE), version 5.0.

### Ethical committee approval

The study was approved by the local Ethical Committee (Comitato Etico Milano Area 2, OSMAMI-19/09/2022-0043481-U) and patients gave informed consent in accordance with the Helsinki Declaration. The histopathological part of the study was retrospective, while the scRNAseq one was prospective. Patients gave informed consent to participate into the registry of AIHA active at our center which includes bone marrow evaluation and collection biological samples (CYTOPAN).

### Bone marrow evaluation

Bone marrow trephines were reviewed by an experienced hematopathologist; BM cellularity was assessed and categorized as normal, increased, or reduced as corrected per age. BM fibrosis was classified according to WHO 2016;^15^ lymphoid infiltrate was evaluated in BM trephines and by cytofluorometry of BM aspirate. Classic cytogenetics was also performed.

For statistical analysis, Student t test was used for continuous variables and chi-square test for categorical ones. Analysis of variance was performed by using mean, median, ranges and standard errors.

## Single cell RNA sequencing

### Sample processing

Nine patients with primary wAIHA belonging to the original cohort were selected for scRNA-seq evaluation on BM aspirate.^28^ These included *n* = 5 patients sampled at diagnosis, *n* = 3 of whom re-sampled at remission at 6 months from diagnosis (*n* = 2 refused to undergo a further BM aspirate), and *n* = 4 patients evaluated at relapse. Mononuclear cells (MNCs) were isolated by Ficoll-Paque medium (Cytiva, Cat# 17144002) density gradient centrifugation.

### 5’ Single-cell RNA sequencing

MNCs were washed thrice with 1X Phosphate-Buffered Saline (PBS, free of calcium and magnesium) containing 0.04% weight/volume FBS (Gibco, Cat# 70011-044). Thereafter MNCs were counted by the Burker chamber. The Chromium Next GEM Single Cell 5’ v2 protocol (CG000330 Rev E) was used to calculate the volume of cells to load for each sample, with the aim of recovering 9000 cells. The Chromium Next GEM Chip K (10X Genomics; PN-2000286) with Master Mix from Chromium Next GEM Single Cell 5’ Reagent Kits v2 (Dual Index) (10X Genomics; PN-1000263) and Chromium Single Cell Human TCR Amplification Kit, (10X Genomics; PN-1000252) were employed to generate Single-cell 5′ RNA-seq libraries. After the GEM was generated and the barcoded full-length cDNA amplificated, the SPRIselect Reagent (Cat# B23318) was used to purify cDNA. The latter was then quantified by Picogreen dsDNA assay kit (Cat# P7589). To assess quality, the Agilent 4150 TapeStation machine was employed by using High Sensitivity D5000 ScreenTape (Agilent, Cat# 5067-5592) with High Sensitivity D5000 Reagents (Agilent, Cat# 5067-5593). Thereafter, Illumina R2 sequence, P5, P7, i5 and i7 sample indexes were added as per standard protocol procedure (10X Genomics; Library Construction Kit, PN- 1000190 and Dual Index Kit TT Set A, PN-1000215, respectively). For TCR analysis, parallelly Chromium Single Cell Human TCR Amplification Kit (10X Genomics; PN-1000252) was utilized. Finally, the quality of libraries was assessed, and the sequencing was performed on a NovaSeq6000 platform (Illumina sequencing system) by utilizing a 150 bp Paired End protocol, which targets approximately 100,000 reads/cell and 15,000 reads/cell for 5’ gene expression and TCR enriched libraries, respectively, as per manufacturer’s recommendations.

### Single-cell RNA sequencing: pre-processing

The gene expression (GEX) and TCR single-cell RNA-seq data were aligned and quantified by employing the Cell Ranger (v 5.0) pipeline (https://www.10xgenomics.com/) against the human genome GRCh38. Eventual ambient RNA contamination was removed by using the SoupX pipeline.^29^ The Seurat pipeline (v 4.4.0) was used to assess expression matrices, and low-quality cells, defined as mitochondria gene content >5%, or number of detected genes <200 or >3000, were filtered out as did doublets by employing the DoubletFinder tool (v 2.0.4); *nCounts_RNA*, *n_FeatureRNA* and *percent_MT* were evaluated for each sample **(**Supplementary Fig. 1). An automated cell assignment was used on filtered single-cell data which was projected on Bone Marrow reference map (v4.4.0, https://satijalab.org/seurat/articles/multimodal_reference_mapping.html) and on a reference atlas for T cells (https://github.com/carmonalab/ProjecTILs).^19^

### Single-cell RNA sequencing data analysis

ScRNAseq analysis was completed by Seurat pipeline (v 4.4.0). After normalizing the counts, the *FindTransferAnchors* and the *IntegrateData* functions with RPCA method were used to perform the integration process. Thereafter, the integrated Seurat object was scaled, and the *FindVariableFeatures* function was employed to obtain the most variable features. Following the *runPCA* function, the matrix was used to build uniform manifold approximation and projection (UMAP); *runPCA* function was then employed to perform the principal components (PCs) analysis basing on these genes. To perform Batch effect correction to PCs Harmony (v.1.2.0) was used^30^ with function *RunHarmony* by utilizing the batch of sequencing as variable for correction. The harmonized matrix was thus used to plot the UMAP. For the polyclonal BM scRNA-seq data set, dimensional reduction and cell clustering were performed by using 30 PCs. The resolution parameter was 0.1. FindMarkers function from Seurat package (v 4.4.0) was employed to perform differential expression analysis between different clinical stages and *FindAllMarkers* and *FindMarkers* functions were utilized to cluster specific markers. Violin plots to compare expression across cell subsets/clinical groups were performed by VlnPlot function in R.

Functional enrichment analysis was made with genes by using the clusterProfiler package (v. 4.10.0), for hallmark gene sets available in the Molecular Signature Database (MSigDB) (msigdbr, v.7.5.1). For all analyses, Wilcox test with a maximum adjusted *p* value Cutoff = 0.1 was employed (Benjamini-Hochberg method was used for *p*-value correction). Figures were plotted by ggplot2 (v 3.4.4) in R. The data of the TCR repertoire were analyzed by using the scRepertoire v.2 package, to explore clones by *clonalQuant, clonalAbundance, clonalLenght* and *clonalCompare* functions.^31^

### Cytosig

Cytokine expression and activity was determined by means of the CytoSig Software for single cell analysis^32^ from matrices selected for previously identified immune cells. Cytokine activity was inferred by CytoSig tool, following the indications available at https://github.com/data2intelligence/CytoSig. Output files included a correlation coefficient matrix, a standard error matrix and a z-score matrix. Comparisons across patient groups were performed through Wilcox test of z-scores of given cytokines. Statistical significance was set at *p* < 0.01 with false discovery rate correction for multiple hypothesis testing.

### Data visualization

All plots were generated using the ggplot2 (v 3.4.4) and ComplexHeatmap (v 2.18.0) packages in R. Data from all other analyses were visualized using GraphPad Prism 10.0. Figures were produced using Adobe Illustrator 2024. Graphical Abstract was created with BioRender.com.

Details on statistical tests used in the different figures and definition of relevant summary statistics are included in the figure legends.

## Supplementary information

Supplementary information

Supplementary tables and figures

## Data Availability

All data have been included in the manuscript. The scRNAseq data have been uploaded in NCBI’s Gene Expression Omnibus and are accessible through GEO Series accession number GSE301528 to a public repository and may be obtained upon request to the corresponding author. Further information may be obtained upon reasonable request to the corresponding author.
