# Supplementary material for: Bone marrow microenvironment in autoimmune hemolytic anemia: from trephine biopsy to single cell RNA sequencing
Source: Signal Transduct Target Ther. 2025 Aug 25;10:277. doi: 10.1038/s41392-025-02348-y (PMC12379653; doi:10.1038/s41392-025-02348-y)
Supplement: Supplementary file 1 — Supplementary information [file 41392_2025_2348_MOESM1_ESM.pdf]

Fondazione IRCCS Ca' Granda
Ospedale Maggiore Policlinico

Sistema Socio Sanitario
Regione Lombardia

Dipartimento di Medicina Interna
UOC EMATOLOGIA - Direttore: Prof. Luca Baldini
Pad. Marcora / Granelli
Direzione Tel.: 02 5503-3422/3342
Amb: 02 5503-3345 - DH: 02 5503-3314
Reparti: CTMO 02 5503-3335 - AOE 02 55033429
Mail: luca.baldini@unimi.it

Evaluation of the diagnostic and therapeutic pathway in patients with autoimmune cytopenia (autoimmune hemolytic anemia, autoimmune thrombocytopenia, and autoimmune neutropenia) and identification of predictive and prognostic markers

Acronym: CYTOPAN

Promotor: Fondazione IRCCS Ca' Granda Ospedale Maggiore Policlinico,
Via Sforza 28, 20122 Milano, Italia

Coordinating centre: U.O. C. Ematologia
Fondazione IRCCS Ca' Granda Ospedale Maggiore Policlinico,
Via Sforza 35, 20122 Milano, Italia

Principal investigator: Dr. Bruno Fattizzo

Signature: ______________________

Version: 2.0

Date: 05 Luglio 2022

Confidentiality Statement
All information contained in this document shall be considered confidential and shall remain the exclusive property of Fondazione IRCCS Ca' Granda Ospedale Maggiore Policlinico. The use of such confidential information is restricted to the recipient for the agreed purpose and must not be disclosed, published, or otherwise communicated to unauthorized individuals, for any reason or in any form, without the prior written consent of Fondazione IRCCS Ca' Granda Ospedale Maggiore Policlinico.

Dipartimento di Medicina Interna
UOC EMATOLOGIA - Direttore: Prof. Luca Baldini
Pad. Marcora / Granelli
Direzione Tel.: 02 5503-3422/3342
Amb: 02 5503-3345 - DH: 02 5503-3314
Reparti: CTMO 02 5503-3335 - AOE 02 55033429
Mail: luca.baldini@unimi.it

LIST OF ABBREVIATIONS

CE: Ethics Committee

CI: Informed Consent

CRF: Case Report Form (Data Collection Form)

GCP: Good Clinical Practice

AIHA: Autoimmune Hemolytic Anemia

ITP: Immune Thrombocytopenia (formerly Idiopathic Thrombocytopenic Purpura)

CIN: Chronic Idiopathic Neutropenia

1. INTRODUCTION

1.1 Background and Rationale

Autoimmune cytopenias, including autoimmune hemolytic anemia (AIHA), immune thrombocytopenia (ITP), and chronic idiopathic neutropenia (CIN), are rare and benign hematological conditions characterized by the production of autoantibodies or the development of autoreactive T lymphocyte clones capable of attacking circulating blood cells (red blood cells, platelets, and neutrophils) or inducing premature death of precursors in the bone marrow (ineffective erythropoiesis). Autoimmune cytopenias represent a spectrum of disease ranging from mild, often asymptomatic forms diagnosed incidentally, to severe and potentially fatal forms. The latter typically manifest acutely with symptoms of severe anemia (fatigue, shortness of breath, anginal pain, etc.), thrombocytopenia (mucosal or cutaneous bleeding of varying severity), or neutropenia (acute, chronic, or recurrent infections). In addition to the direct consequences of immune cytopenias, associated complications related to the disease or therapies may occur, including thrombosis, infections, and acute or chronic renal failure. Moreover, the three cytopenias may be variably associated, forming Evans syndrome.

Currently, the main predictor of severity, relapse, and onset of complications used by clinicians is the severity of cytopenia at onset: for example, patients with severe anemia (Hb <6 g/dL) have approximately double the risk of relapse compared to those with moderate or mild anemia. Another known risk factor for relapse is the presence of Evans syndrome. Infectious complications and acute renal failure correlate with mortality, which for autoimmune hemolytic anemia is estimated to be around 10%.

Despite this limited evidence, the rarity of these diseases and the lack of adequate prospective studies make prognostic assessment and management—especially during the two most critical phases, onset (especially if acute) and severe relapses requiring multiple therapy lines and hospitalization—difficult. During these phases, it may be necessary to tailor the aggressiveness of therapy and potential transfusional support based on numerous clinical and laboratory data that are not yet standardized. For example, it is currently unknown which patients should receive anticoagulant prophylaxis, except

Dipartimento di Medicina Interna
UOC EMATOLOGIA - Direttore: Prof. Luca Baldini
Pad. Marcora / Granelli
Direzione Tel.: 02 5503-3422/3342
Amb: 02 5503-3345 - DH: 02 5503-3314
Reparti: CTMO 02 5503-3335 - AOE 02 55033429
Mail: luca.baldini@unimi.it

for those with known additional risk factors (diabetes, obesity, factor V Leiden or prothrombin mutation, etc.).

On the other hand, the availability of new drugs for autoimmune cytopenias (rituximab, thrombopoietin receptor agonists, complement inhibitors, fostamatinib, neonatal Fc receptor inhibitors, and tyrosine kinase inhibitors) makes it even more necessary to identify ideal candidates for treatment and predictive factors for relapse, non-response, and complications, particularly infections. This would improve therapeutic choices and monitoring of complications.

Finally, some patients with isolated cytopenia show bone marrow features such as dysmyelopoiesis and clonal cell populations (myeloid or lymphoid), potentially evolving into overt hematologic malignancies. Currently, there are no tools to predict this evolution risk, and studying somatic DNA mutations via targeted sequencing could provide insights.

From a diagnostic standpoint, the list of tests to use in each autoimmune cytopenia is not standardized, requiring continuous exchange of information between the clinician in charge (e.g., internal medicine specialist) and the hematologist. A critical evaluation of the tests used, their sensitivity and specificity, and the discharge diagnoses of hospitalized cytopenic patients could provide important information for diagnostic pathway rationalization.

Based on these considerations, AIHA, ITP, and CIN require more standardized approaches, both in routine tests (CBC, hemolysis indices, autoimmunity markers, electrophoresis, etc.) and experimental markers such as somatic DNA mutations, levels of specific immunomodulatory cytokines, lymphocyte subpopulations, and the microbiota. The microbiota, which comprises the microbial flora colonizing specific body districts (e.g., respiratory and intestinal tracts), is increasingly recognized as an active player in autoimmune disease pathogenesis and complications. Studying its complexity at diagnosis and various time points during treatment could provide crucial information on relapse risk and especially infection risk in these often heavily immunosuppressed patients.

The aim of this project is to critically evaluate the diagnostic and therapeutic pathway of patients with autoimmune cytopenia, particularly those hospitalized at this hospital, to create a list of diagnostic tests appropriate for different clinical levels and identify prognostic and predictive markers for relapse, therapeutic response, and complications including death. Secondarily, it will evaluate whether the study of somatic DNA mutations, cytokine profiles, lymphocyte subpopulations, and respiratory and intestinal microbiota can serve as valid and accurate tools to provide information on disease severity, relapse tendency, and infection risk. If this working hypothesis proves valid, it could justify exploring these parameters at diagnosis and during treatment, potentially developing dynamic biomarkers anticipating relapse or severe infections.

---

2. STUDY OBJECTIVES

2.1 Primary Objective

- Characterize the diagnostic pathway of newly diagnosed autoimmune cytopenia patients hospitalized or attending the Hematology outpatient clinics of this hospital.

Dipartimento di Medicina Interna
UOC EMATOLOGIA - Direttore: Prof. Luca Baldini
Pad. Marcora / Granelli
Direzione Tel.: 02 5503-3422/3342
Amb: 02 5503-3345 - DH: 02 5503-3314
Reparti: CTMO 02 5503-3335 - AOE 02 55033429
Mail: luca.baldini@unimi.it

- Determine sensitivity and specificity of the tests used and prepare a list of recommended diagnostic tests for autoimmune cytopenia at various clinical levels.

2.2 Secondary Objectives

- Evaluate clinical and laboratory markers predictive of therapeutic response and complications, particularly infections, through longitudinal collection of clinical data, serum/plasma/bone marrow samples, nasopharyngeal swabs, and fecal samples during the observational study period.
- Preliminary correlation analysis between identified somatic DNA mutations, cytokine patterns, microbiota complexity at diagnosis and during disease course with therapeutic response, relapses, and complications (especially infections).
- Evaluate red blood cell metabolism in a subgroup of patients with cytopenias and bone marrow dysplasia and measure in vitro activity of glycolysis-activating drugs (specific substudy objective).

---

3. STUDY DESIGN

3.1 Design

The study will enroll patients with anemia (Hb <12 g/dL), thrombocytopenia (platelets <100,000/mm^3), and neutropenia (neutrophils <1000/mm^3) without prior hematologic diagnosis, hospitalized or attending Hematology outpatient clinics. Diagnosis of autoimmune cytopenia will follow current accepted criteria for AIHA (evidence of hemolysis and positive/negative direct Coombs test after exclusion of other hemolysis causes), ITP (exclusion of other thrombocytopenia causes, with or without anti-platelet antibodies), and CIN (exclusion of other neutropenia causes, with or without anti-neutrophil antibodies).

To ensure informative clinical follow-up on test sensitivity and specificity and to establish a control group, all newly diagnosed cytopenia patients without known primary cause will be enrolled. Patients will be classified by severity and, for AIHA, by antibody characteristics:

- Moderate ITP: platelets >30,000/mm^3
- Severe ITP: platelets <30,000/mm^3
- Mild neutropenia: neutrophils >1000/mm^3
- Moderate neutropenia: neutrophils 500–1000/mm^3
- Severe neutropenia: neutrophils <500/mm^3
- Warm AIHA: monospecific direct Coombs positive for IgG or IgG+C low titer, no autoagglutination at 20°C
- Cold AIHA: monospecific direct Coombs positive for C3d fraction and/or cold agglutinins with or without autoagglutination at 20°C

Dipartimento di Medicina Interna
UOC EMATOLOGIA - Direttore: Prof. Luca Baldini
Pad. Marcora / Granelli
Direzione Tel.: 02 5503-3422/3342
Amb: 02 5503-3345 - DH: 02 5503-3314
Reparti: CTMO 02 5503-3335 - AOE 02 55033429
Mail: luca.baldini@unimi.it

- Mixed AIHA: monospecific direct Coombs positive for high-titer IgG+C and autoagglutination at 20°C and/or dual specificity for anti-Rh and anti-I/i/IH
- Atypical AIHA: monospecific direct Coombs positive for IgA, for warm IgM, or direct Coombs negative after exclusion of other hemolysis causes.

Availability of clinical information at diagnosis and follow-up updates for at least 3 years are key inclusion criteria.

3.2 Inclusion Criteria
Patients fulfilling all the following:

- New diagnosis of cytopenia without known secondary cause
- Male or female ≥18 years old
- Able to provide informed consent
- Willing to undergo regular clinical follow-up for 3 years after enrollment

For substudy participation, additional criteria:

- Bone marrow evaluation.

3.3 Exclusion Criteria
Patients with any condition potentially interfering with protocol adherence or informed consent.

---

4. STUDY PROCEDURES
4.1 Intervention
Enrollment:

- Venous blood draw for somatic DNA mutation and cytokine profile studies
- Nasopharyngeal swab and fecal sample for microbiota analysis
- Medical history, physical exam, anthropometrics
- Blood, urine, and instrumental tests as clinically indicated to assess hematologic disease status
- For the marrow substudy, samples will be used to study immunological microenvironment, cytokine patterns, and RBC metabolism.

Follow-up (every 1–3 months for AIHA and ITP, every 6–12 months for CIN, according to clinical need):

- Blood for cytokine profiling
- Nasopharyngeal swab and fecal sample for microbiota analysis

Dipartimento di Medicina Interna
UOC EMATOLOGIA - Direttore: Prof. Luca Baldini
Pad. Marcora / Granelli
Direzione Tel.: 02 5503-3422/3342
Amb: 02 5503-3345 - DH: 02 5503-3314
Reparti: CTMO 02 5503-3335 - AOE 02 55033429
Mail: luca.baldini@unimi.it

- Medical history, physical exam, anthropometrics
- Blood, urine, instrumental tests as indicated

At relapse:

- Blood/marrow, nasopharyngeal swab, fecal sample, clinical evaluation, and lab tests as above. Samples stored at the Hematology and Transfusion Medicine Laboratory biobank.

5. ENDPOINT

5.1 Primary endpoint

Number of laboratory tests required to reach the diagnosis of autoimmune cytopenia for each patient.

5.2 Secondary endpoints

Type of laboratory tests required to reach the diagnosis of autoimmune cytopenia and to investigate physiopathology of the disease.
Number and type of imaging tests required to reach the diagnosis of autoimmune cytopenia.

6. STUDY DURATION/TIMELINE

Enrollment is expected to be completed within 2 years.
All patients included in the study will have a minimum follow-up of 3 years.
At the end of the enrollment phase, it will be evaluated whether the sample size is consistent with the preliminary enrollment estimates. Otherwise, enrollment will be extended by an additional 12 months.

7. EXPERIMENTAL DESIGN

7.1 Sample size

On average, our Center annually evaluates 40 new patients with autoimmune hemolytic anemia (AIHA), 40 with immune thrombocytopenia (ITP), and 20 with cytopenia of undetermined significance (CIN). Therefore, enrollment of at least 200 patients is expected. Considering that the relapse risk is approximately 30% in AIHA and ITP and most relapses occur within the first 24 months from onset, it is reasonable to estimate that about 40-50 patients will experience relapse during the three-year follow-up.

7.2 Procedures

All procedures will be conducted at the laboratory of the UOS Anemia Pathophysiology, Mangiagalli Pavilion ground floor, part of the UOC Hematology.

Molecular profiling analysis

Somatic mutations in genes reported to be associated with myeloid pathology potentially linked to the pathogenesis or clinical behavior of autoimmune cytopenias will be analyzed. Whole exome sequencing (WES) will be performed, focusing first on genes involved in proliferation/apoptosis pathways (RAS, TP53), DNA methylation (IDH1/IDH2, DNMT3A, TET2, ASXL1), and RNA splicing (SF3B1, NOTCH), and then extended to possible pathogenic variants. WES analysis will be based on a target enrichment system for next generation sequencing (NGS), allowing efficient, simple, and rapid analysis of genomic regions of interest in a large number of samples. For NGS, 50 ng of non-amplified genomic DNA from peripheral blood mononuclear cells will be enriched for target exons by liquid-

Dipartimento di Medicina Interna
UOC EMATOLOGIA - Direttore: Prof. Luca Baldini
Pad. Marcora / Granelli
Direzione Tel.: 02 5503-3422/3342
Amb: 02 5503-3345 - DH: 02 5503-3314
Reparti: CTMO 02 5503-3335 - AOE 02 55033429
Mail: luca.baldini@unimi.it

phase hybridization using a SureSelect custom kit designed to capture all coding exons (n = 2,045) of 104 target genes (Agilent Technologies), according to an optimized automated processing protocol. After attaching index sequences to discriminate respective samples, the enriched target DNA will undergo massive parallel sequencing using a NextSeq 550 sequencer (Illumina) with a default protocol of 150 bp paired-end reads. Sequencing results will be analyzed through our pipeline to identify somatic mutations. All sequencing reads will be aligned to the human reference genome (hg19) using BWA version 0.5.8 with standard parameters. After removing duplicate or low-quality reads, allele frequencies of single nucleotide variants (SNVs) and indels at each genomic position will be calculated by enumerating relevant reads using samtools. Variants with allele frequency >2% and supported by >5 reads will be extracted and annotated using ANNOVAR28. Synonymous and ambiguous variants will be discarded.
Missense SNVs already present in public and private single nucleotide polymorphism (SNP) databases and missense SNVs with allele frequency between 0.45 and 0.55 will be filtered as polymorphisms. Among missense SNVs with allele frequency 0.45–0.55, known hotspot mutations will be considered somatic mutations. The significance of observed mutations in each gene will be assessed by calculating the type I error under the null hypothesis that all observed non-silent mutations are "passenger changes," assuming a Poisson distribution with a uniform background mutation rate (λ).

Cytokine profile analysis
The following cytokines will be evaluated: interleukin (IL)-6, IL-10, IL-17, tumor necrosis factor (TNF)-alpha, interferon (IFN)-gamma, and transforming growth factor (TGF)-beta. Serum samples from patients and control donors matched for sex and age will be tested. Measurements will be performed using commercial ELISA kits (i.e., High Sensitivity ELISA kits, Invitrogen by Thermo Fisher Scientific, MA, USA, human TGF-beta ELISA kit, Immunological Sciences, Rome, Italy), and by single cell RNA sequencing.

Microbiota analysis
For each enrolled case, a nasopharyngeal swab and a fecal sample will be collected at enrollment and at any relapse, prior to the administration of any new line of therapy. Respiratory/fecal samples collected will undergo bacterial genomic DNA extraction and amplification of the hypervariable V1–V3 region of the 16S rRNA gene. Library preparation and subsequent sequencing using the MiSeq Illumina method will enable precise characterization of the resident flora, at least at the bacterial genus level. Data will be analyzed with appropriate statistical methods to evaluate potential differences in nasal/fecal microbiota composition during follow-up and in relation to different therapies administered.

Red blood cell metabolism analysis
In the subgroup of patients with cytopenia and bone marrow dysplasia, enzymatic activity of glycolytic enzymes will be studied on an aliquot of the blood sample collected at enrollment, and activity of some glycolysis-activating drugs may be measured in vitro. No genetic studies will be performed. The aliquot will be taken from the main study blood sample and used to determine the activity of red blood cell glycolytic chain enzymes. Glycolytic activity will be measured at baseline and after 6-hour in vitro incubation with AG946.

Dipartimento di Medicina Interna
UOC EMATOLOGIA - Direttore: Prof. Luca Baldini
Pad. Marcora / Granelli
Direzione Tel.: 02 5503-3422/3342
Amb: 02 5503-3345 - DH: 02 5503-3314
Reparti: CTMO 02 5503-3335 - AOE 02 55033429
Mail: luca.baldini@unimi.it

Statistical analysis

Since the study has a purely descriptive purpose, aimed at understanding the distribution and parameters of the variable under study (number of diagnostic tests per patient), and no data are available in the literature on this matter, the sample size is estimated based on the number of newly diagnosed autoimmune cytopenia patients expected to be diagnosed in one year at the proposing UOC (100 new cases/year; 200 total new cases considering the 2-year enrollment period), autoimmune cytopenia being a rare disease with estimated prevalence of 1-3 new cases per 10^5 (Barcellini et al., Blood 2014).

The diagnostic utility of performed tests will be evaluated by calculating their sensitivity and specificity, with particular reference to the definitive hematologic diagnosis in patients hospitalized in a ward.

The cytokine profile and microbiota identified at diagnosis and at follow-up points or relapses will be correlated with clinical outcomes, measured in terms of response to specific therapy, relapse-free survival (RFS), defined as the time interval between response to a given therapy and relapse, and the occurrence of main complications (thrombosis, acute renal failure, and infections). RFS curves will be constructed using the Kaplan-Meier method and compared by the Log-rank test or Cox proportional hazards model.

Expected results

The study aims to establish a list of diagnostic tests to propose as a clinical workup in patients with suspected newly diagnosed autoimmune cytopenia, particularly those hospitalized.

A major goal is to evaluate whether a cytokine profile or microbiome characteristics can be used to infer response to a given therapy, risk of relapse, and the occurrence of disease or treatment-related complications, especially infections.

Specifically, this pilot study will enable:

- Development of cytokine profiling methodology for patients with newly diagnosed immune cytopenia;
- Definition of the microbiological repertoire of the microbiome in patients with AEA, ITP, and CIN, previously uncharacterized;
- Preliminary evaluation of correlations between cytokine profile, fecal and respiratory microbiome at diagnosis or during follow-up, and clinical outcome of patients included in the study.

This may allow identification of immunological and microbiological profiles contributing to therapy response, relapse, and potentially fatal complications in patients with AEA, ITP, and CIN, enabling more appropriate therapy and prophylaxis choices.

8. ADVERSE EVENTS

The project does not involve administration of drugs or other substances nor invasive clinical practices. Therefore, no adverse events are expected.

Dipartimento di Medicina Interna
UOC EMATOLOGIA - Direttore: Prof. Luca Baldini
Pad. Marcora / Granelli
Direzione Tel.: 02 5503-3422/3342
Amb: 02 5503-3345 - DH: 02 5503-3314
Reparti: CTMO 02 5503-3335 - AOE 02 55033429
Mail: luca.baldini@unimi.it

9. RISK/BENEFIT ASSESSMENT

Collected data will enable a more rational choice of diagnostic tests to perform in a patient with autoimmune cytopenia. Analysis of blood samples, respiratory and fecal swabs may help better understand disease mechanisms and guide future therapeutic choices.

10. STUDY MANAGEMENT

10.1 Data collection and management

A responsible referent will be identified for procedures of data collection, quality verification, and transmission. Collected information will be entered by trained personnel into a dedicated database. At study registration and during scheduled periodic control visits (according to standard clinical practice) or disease progression, specified clinical information and samples of peripheral blood for molecular and cytokine studies, fecal samples, and nasopharyngeal swabs for microbiome studies must be collected.
Registration will be done in a limited-access database: the physician will complete the registration form after obtaining dated and signed informed consent from the patient. Patients will be assigned a progressive three-digit identification code starting from 001 based on enrollment order.

10.2 Regulatory approvals

10.2.1 Competent Authority Approval

In accordance with current regulations, the principal investigator must obtain approval from the appropriate Competent Authority before starting the clinical study.
This study will be conducted in compliance with ICH/GCP guidelines and all applicable laws, including the Declaration of Helsinki (June 1964), as amended by the latest World Medical Association General Assembly in Seoul, 2008.

10.2.2 Ethics Committee Approval

The investigator must ensure that the protocol has been reviewed and approved by the local independent Ethics Committee (EC) before study initiation.
The EC must also review and approve the informed consent form (ICF) and all written information provided to patients prior to enrollment.
If protocol and/or ICF amendments are necessary during the study, the investigator is responsible for ensuring review and approval of such modifications by the EC before implementation. Until approval, the previous version of the document must be used.

10.2.3 Informed Consent (IC)

The investigator or designated personnel must inform subjects about all aspects and procedures of the study.
The informed consent process must comply with applicable regulatory procedures. The investigator (or designated collaborator) and the subject must date and sign the consent form before any study-related procedures. Subjects receive a copy of the signed IC; the original is kept in designated study archives. Neither investigator nor staff may coerce or unduly influence subjects to participate or continue in the study. Participation must be voluntary, and subjects must be informed they may withdraw consent at any time without penalty or loss of benefits.
Written or oral study information, including consent forms, must not contain language that waives

Dipartimento di Medicina Interna
UOC EMATOLOGIA - Direttore: Prof. Luca Baldini
Pad. Marcora / Granelli
Direzione Tel.: 02 5503-3422/3342
Amb: 02 5503-3345 - DH: 02 5503-3314
Reparti: CTMO 02 5503-3335 - AOE 02 55033429
Mail: luca.baldini@unimi.it

subjects' legal rights or releases investigators or sponsors from liability for negligence.
Patients meeting inclusion criteria will be invited to provide peripheral blood samples for cytokine profiling during scheduled visits and symptom-driven visits, and consent to use of their personal and clinical data for research. Nasopharyngeal swabs and fecal samples will be collected at enrollment and at 1, 3, 6, and 12 months for microbiota complexity studies.
Patients will have adequate time to read the information and ask questions.

10.3 Investigator duties
According to applicable local regulations, the investigator must submit periodic reports on study progress to the EC and notify study closure. These reports and closure notifications are investigator responsibilities.

10.4 Study monitoring
In compliance with applicable regulations and Good Clinical Practice (GCP monitoring visits will be conducted to verify study data accuracy and adherence to protocol and regulatory requirements).

10.5 Quality Assurance of the Study
As the sponsor, Fondazione IRCCS Ca' Granda, Ospedale Maggiore Policlinico may, at its discretion, perform quality control of the study. In this case, the investigator must allow the monitor direct access to all relevant documentation and dedicate part of their time and staff to the auditor to discuss the monitoring results and any other aspects of the study.
Furthermore, Regulatory Authorities may conduct inspections. In this case, the investigator must authorize the inspector direct access to all relevant documentation and dedicate part of their time and staff to the inspector to discuss the monitoring results and any other aspects of the study.

10.6 Study Closure
At the time of study closure, the monitor and investigator must activate a series of procedures:

- review all study documentation
- reconcile the study data
- reconcile all query reports.

10.7 Document Archiving
In compliance with current national regulations, the investigator must keep a copy of all documentation and store it in a dry and secure place after the study closure.

10.8 Disclosure of Information Regarding Scientific Discovery
10.8.1 Confidentiality
The investigator and other personnel involved in the study must treat all information related to the study (including the protocol, obtained data, and all documentation produced during the study) confidentially and must not use such information, data, or reports for purposes other than those described in the protocol. These restrictions do not apply to:

1. information that becomes publicly available, not due to negligence by the investigator or their personnel;

Dipartimento di Medicina Interna
UOC EMATOLOGIA - Direttore: Prof. Luca Baldini
Pad. Marcora / Granelli
Direzione Tel.: 02 5503-3422/3342
Amb: 02 5503-3345 - DH: 02 5503-3314
Reparti: CTMO 02 5503-3335 - AOE 02 55033429
Mail: luca.baldini@unimi.it

2. information that requires confidential disclosure to the Ethics Committee solely for the purpose of evaluating the study;
3. information that must be disclosed to provide adequate medical care to a study subject.

10.8.2 Publications
Fondazione IRCCS Ca' Granda, Ospedale Maggiore Policlinico is the sole owner of the data. The owner of the collected data will be the study sponsor. The scientific responsible of the study will commit to drafting a final report and a scientific article and to making the results public upon study completion. The data will be made public in an anonymized form and presented as aggregated data as required.

11. Indemnity and Compensation in Case of Damage
In case of adverse events or any damage resulting from participation in the research, the Insurance Policy of our Institute also covers participants involved in research projects.

12. Protocol Amendments
In the case of significant modifications to the protocol, the Principal Investigator (PI) commits to notifying the Ethics Committee.

13. Financial Agreements
No financial agreements are foreseen for this study. The study costs will be covered by internal funds of the Hematology Unit originating from the 2020 Current Research budget.
For the substudy on patients with myelodysplasia, Agios Pharmaceuticals will provide the allosteric activator AG946 and finance the purchase of consumables, as detailed in the specific cost form.

14. Disclosure of Conflicts of Interest
The study is sponsored by Fondazione IRCCS Ca' Granda, Ospedale Maggiore Policlinico. The Study Director declares no current conflicts of interest with companies that may be involved in the study, even only as suppliers of materials useful for the study's completion.

15. References
Barcellini W, Fattizzo B, Zaninoni A. Current and emerging treatment options for autoimmune hemolytic anemia. Expert Rev Clin Immunol. 2018 Oct;14:857-872.

Barcellini W, Zaninoni A, Fattizzo B, Giannotta JA, Lunghi M, Ferrari A, Leporace AP, Maschio N, Scaramucci L, Cantoni S, Chiurazzi F, Consonni D, Rossi G, De Fabritiis P, Gaidano G, Zanella A, Cortelezzi A. Predictors of refractoriness to therapy and healthcare resource utilization in 378 patients with primary autoimmune hemolytic anemia from 8 Italian Reference Centers. Am J Hematol. 2018 Jul 7.

Barcellini W, Fattizzo B, Zaninoni A, Radice T, Nichele I, Di Bona E, Lunghi M, Tassinari C, Alfinito F, Ferrari A, Leporace AP, Niscola P, Carpenedo M, Boschetti C, Revelli N, Villa MA, Consonni D, Scaramucci L, De Fabritiis P, Tagariello G, Gaidano G, Rodeghiero F, Cortelezzi A, Zanella A. Clinical heterogeneity and predictors of outcome in primary autoimmune hemolytic anemia: a GIMEMA study of 308 patients. Blood. 2014 Nov 6;124(19):2930-6.

Dipartimento di Medicina Interna
UOC EMATOLOGIA - Direttore: Prof. Luca Baldini
Pad. Marcora / Granelli
Direzione Tel.: 02 5503-3422/3342
Amb: 02 5503-3345 - DH: 02 5503-3314
Reparti: CTMO 02 5503-3335 - AOE 02 55033429
Mail: luca.baldini@unimi.it

Fattizzo B, Zaninoni A, Consonni D, Zanella A, Gianelli U, Cortelezzi A, Barcellini W. Is chronic neutropenia always a benign disease? Evidence from a 5-year prospective study. Eur J Intern Med. 2015 Oct;26(8):611-5.

---

16. Appendix

Not applicable
