# Supplementary material for: Bone marrow microenvironment in autoimmune hemolytic anemia: from trephine biopsy to single cell RNA sequencing
Source: Signal Transduct Target Ther. 2025 Aug 25;10:277. doi: 10.1038/s41392-025-02348-y (PMC12379653; doi:10.1038/s41392-025-02348-y)
Supplement: Supplementary file 2 — Supplementary tables and figures [file 41392_2025_2348_MOESM2_ESM.docx]

Supplementary Materials for

Bone marrow microenvironment in autoimmune hemolytic anemia: from trephine biopsy to single cell RNA sequencing

Bruno Fattizzo^1,2*#^ & Matteo Claudio Da Vià^1*^, Francesca Lazzaroni^1^, Alfredo Marchetti^1,2^,

[Alessio Marella](https://pubmed.ncbi.nlm.nih.gov/?term=Marella%2BA&cauthor_id=38830132)^1^, [Akihiro Maeda](https://pubmed.ncbi.nlm.nih.gov/?term=Maeda%2BA&cauthor_id=38830132)^1^, Antonio Giovanni Solimando^3^, Loredana Pettine^1^, Francesco Passamonti^1,2^, Niccolò Bolli^1,2^ & Wilma Barcellini^1^

Correspondence to: [bruno.fattizzo@unimi.](mailto:xxxxx@xxxx.xxx)it

**This PDF file includes:**

Figures. S1 to S11

Tables S1 to S3


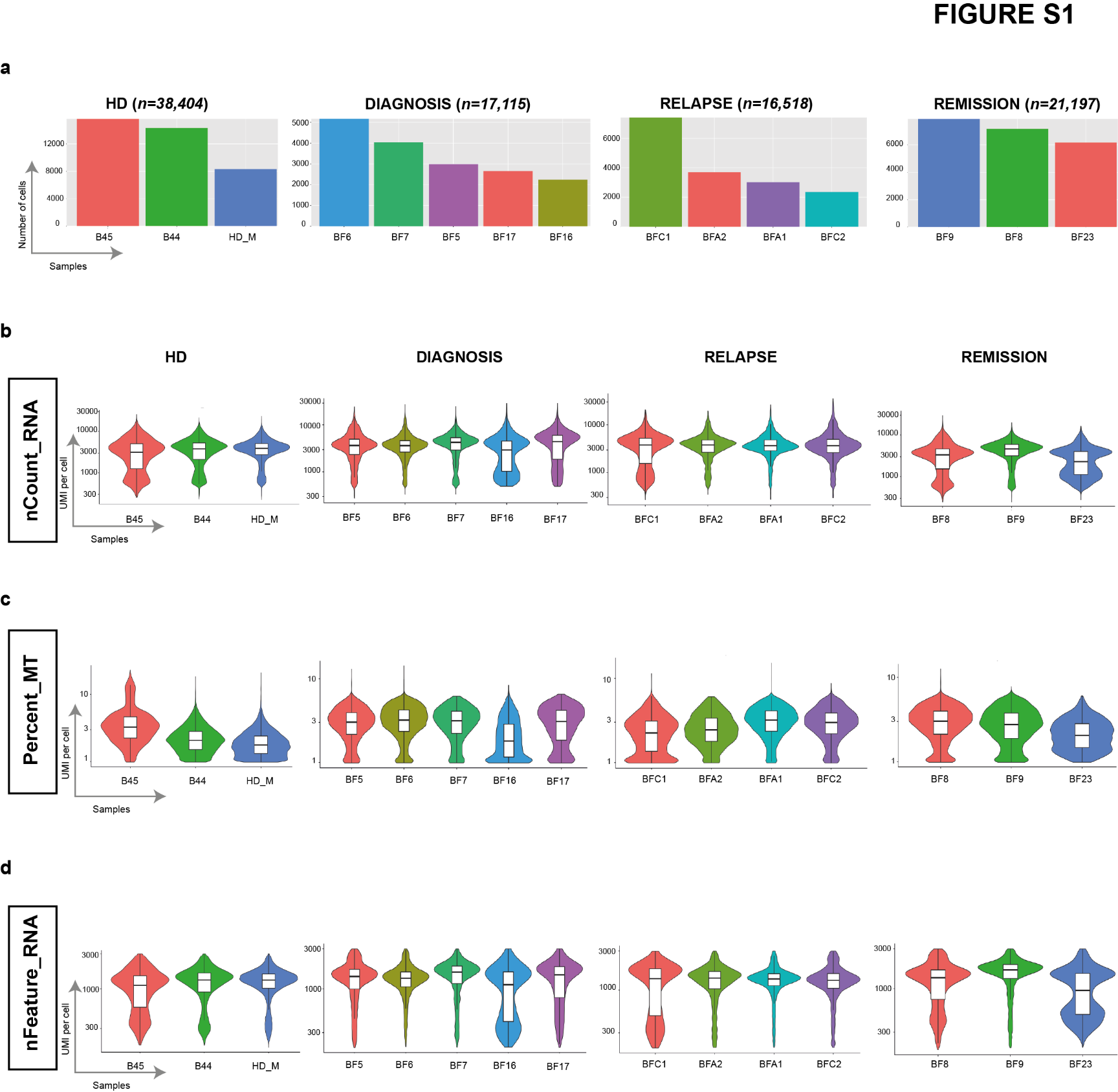


**Figure S1. Quality control and pre-processing of single-cell RNA-seq AHIA data**

Violin plots showing the distribution of key quality control metrics: the number of cells (a), the percentage of “nCounts_RNA” (b), mitochondrial gene percentage (c), and “nFeature_RNA” (d). Data are grouped by diagnosis (relapsed/refractory vs. remission).


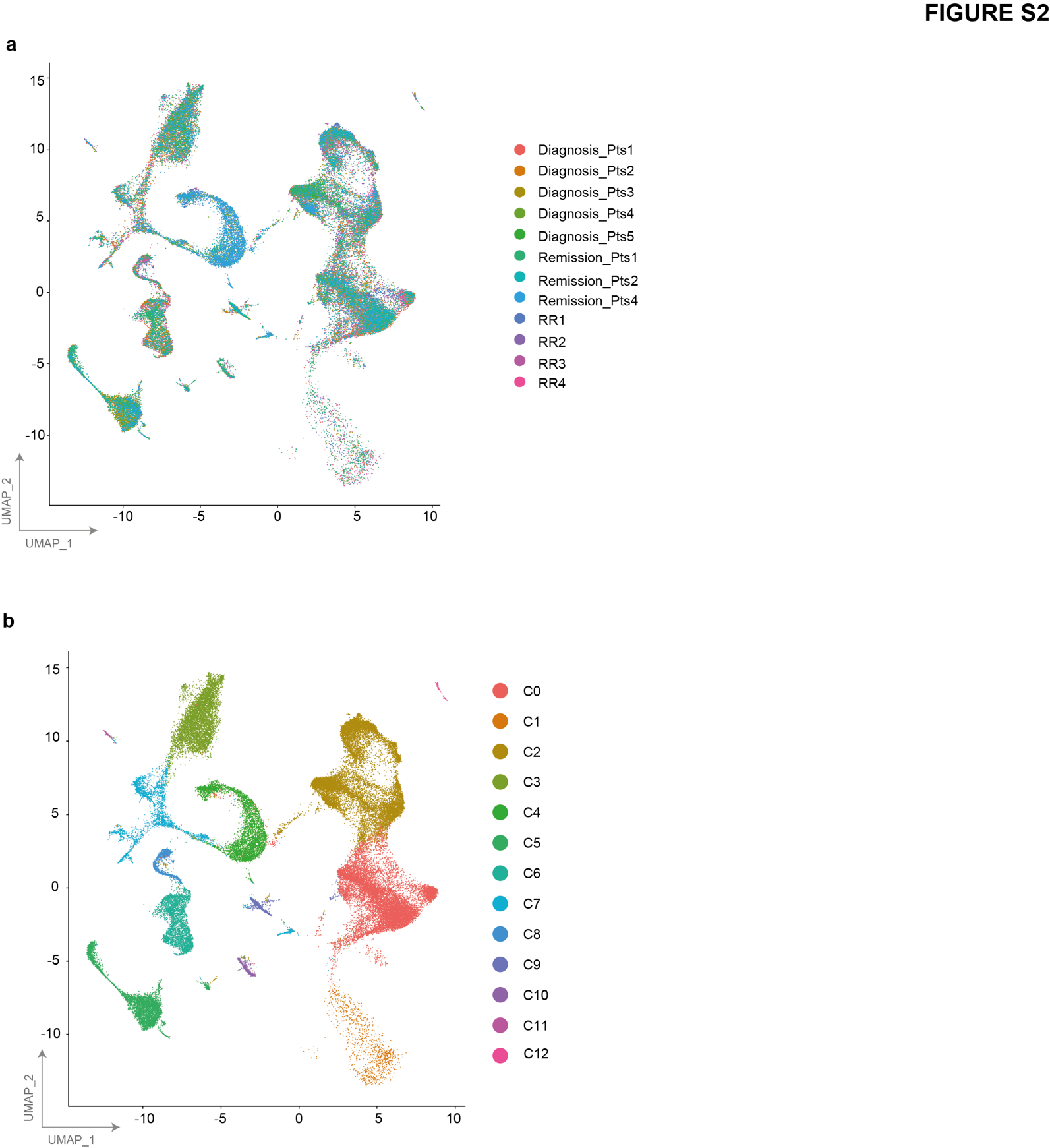


**Figure S2. AHIA MNCs clustering**

(a, b) Uniform Manifold Approximation and Projection (UMAP) of single-cell RNA-seq AHIA data, colored by patient identity (a) and Seurat clusters (b).


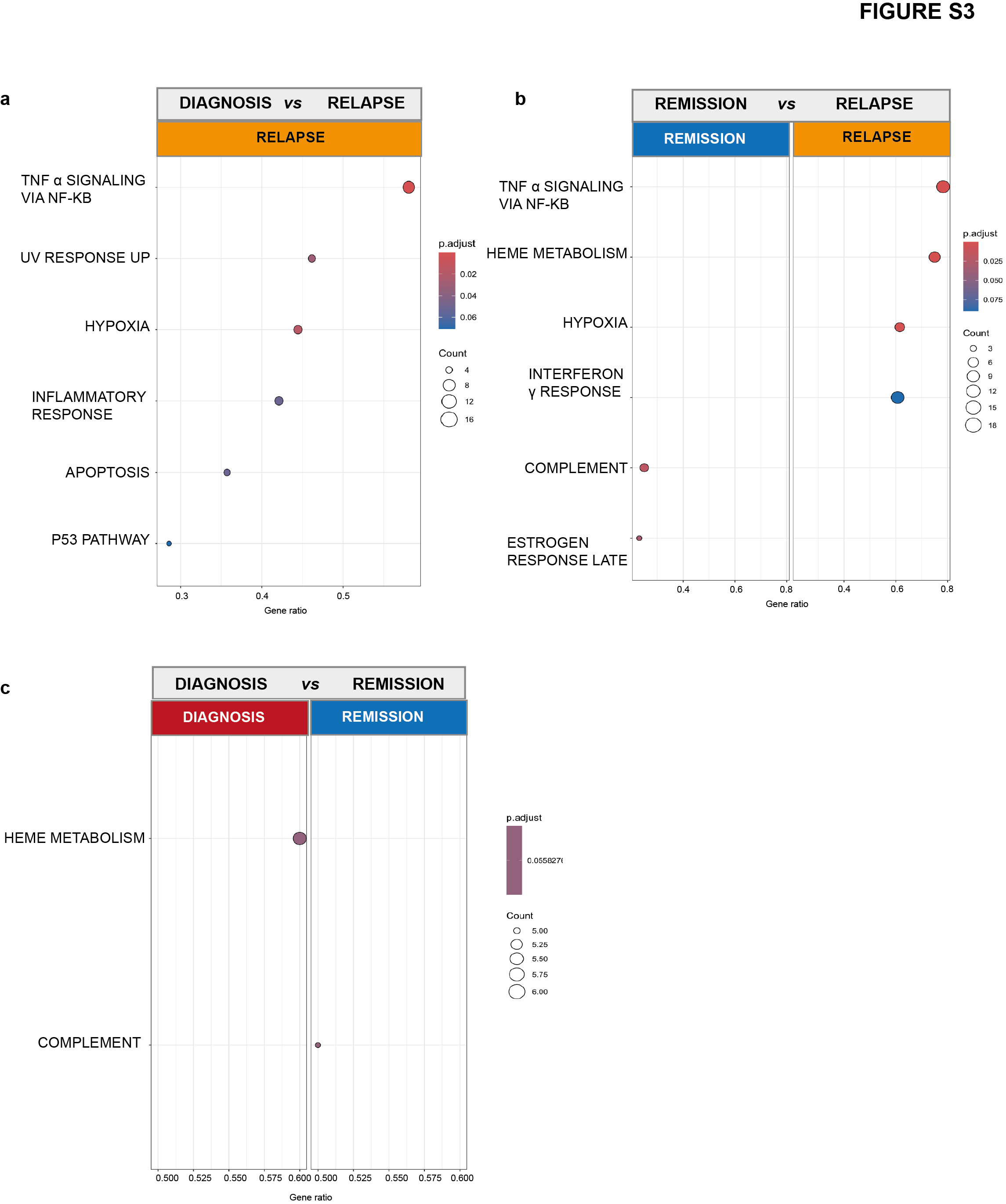


**Figure S3. Hallmark analysis of AHIA single-cell data**

(a-c) Differential expression analysis using Wilcoxon rank-sum test to identify enriched Hallmark gene sets between clinical groups: (a) diagnosis vs. relapsed/refractory (RR), (b) remission vs. RR and diagnosis vs. remission (c). The y-axis shows the gene ratio, with dot size representing counts and color indicating adjusted p-values (padj) in z-score.


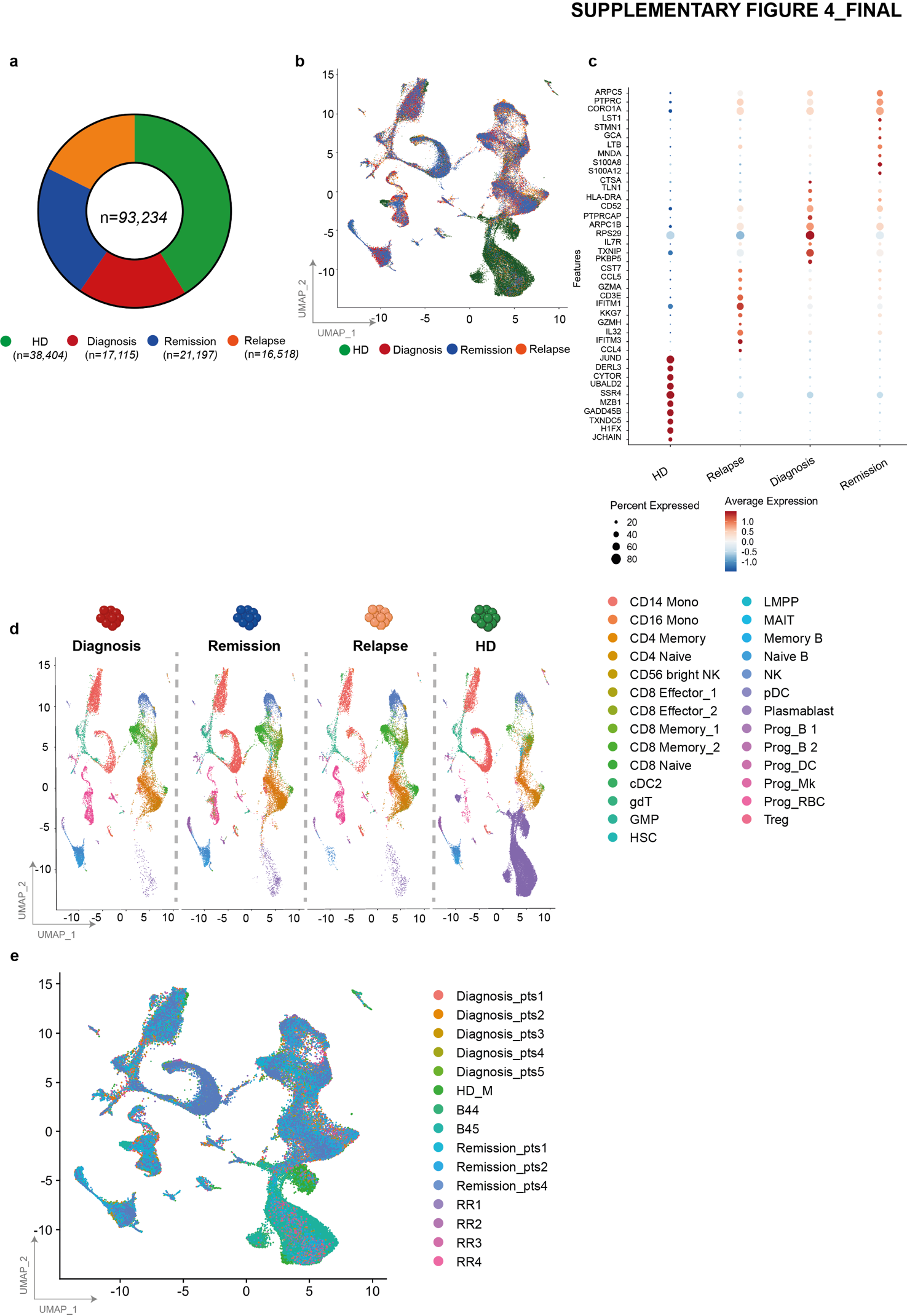


**Figure S4. Single-cell RNA-seq analyses with Healthy Donors**

(a) Donut charts showing the distribution of pathological cells in AHIA patients and healthy donors, stratified by clinical categories. Numerical values for each category are provided in the legend.
(b) UMAP representation of samples colored by clinical stratification, with numerical values and percentages for each category shown in the legend.
(c) Dot plot of the top 15 marker genes distinguishing each clinical state. The x-axis represents clinical categories, and the y-axis lists gene names. Circle size indicates the number of cells expressing the gene, while color intensity reflects gene expression levels.
(d, e) UMAP representations showing cell assignments based on clinical status (d) and by individual patients (e).


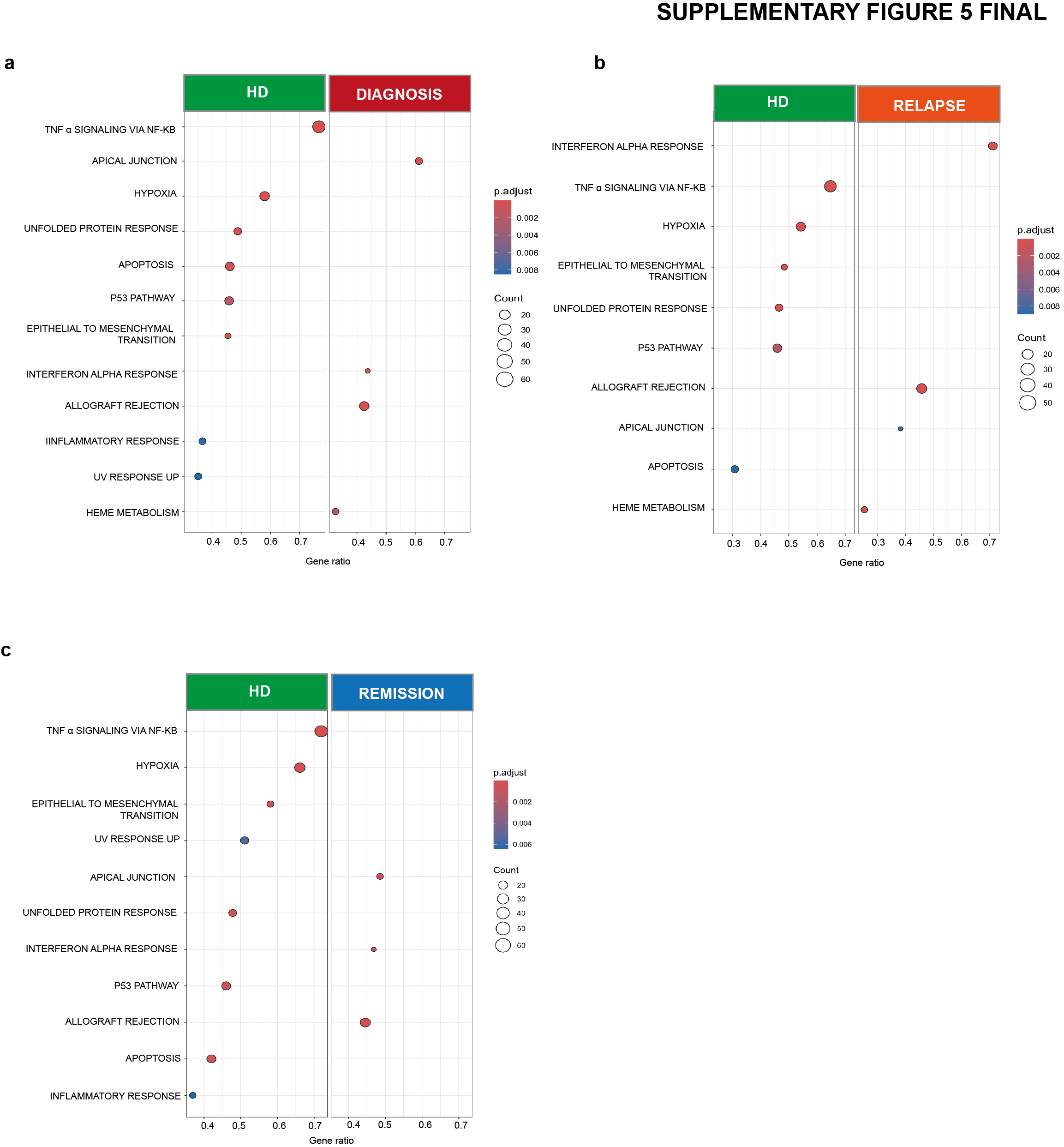
Figure. S5.

**Figure S5. Hallmark analysis of AHIA single-cell data compared to Healthy Donors**
(a-c) Differential expression analysis using the Wilcoxon rank-sum test to identify enriched Hallmark gene sets across clinical groups: (a) Healthy Donors (HD) vs. diagnosis, (b) HD vs. relapsed/refractory (RR), and (c) HD vs. remission. The y-axis displays the gene ratio, with dot size representing the number of counts and color indicating the adjusted p-values (padj) in z-score.


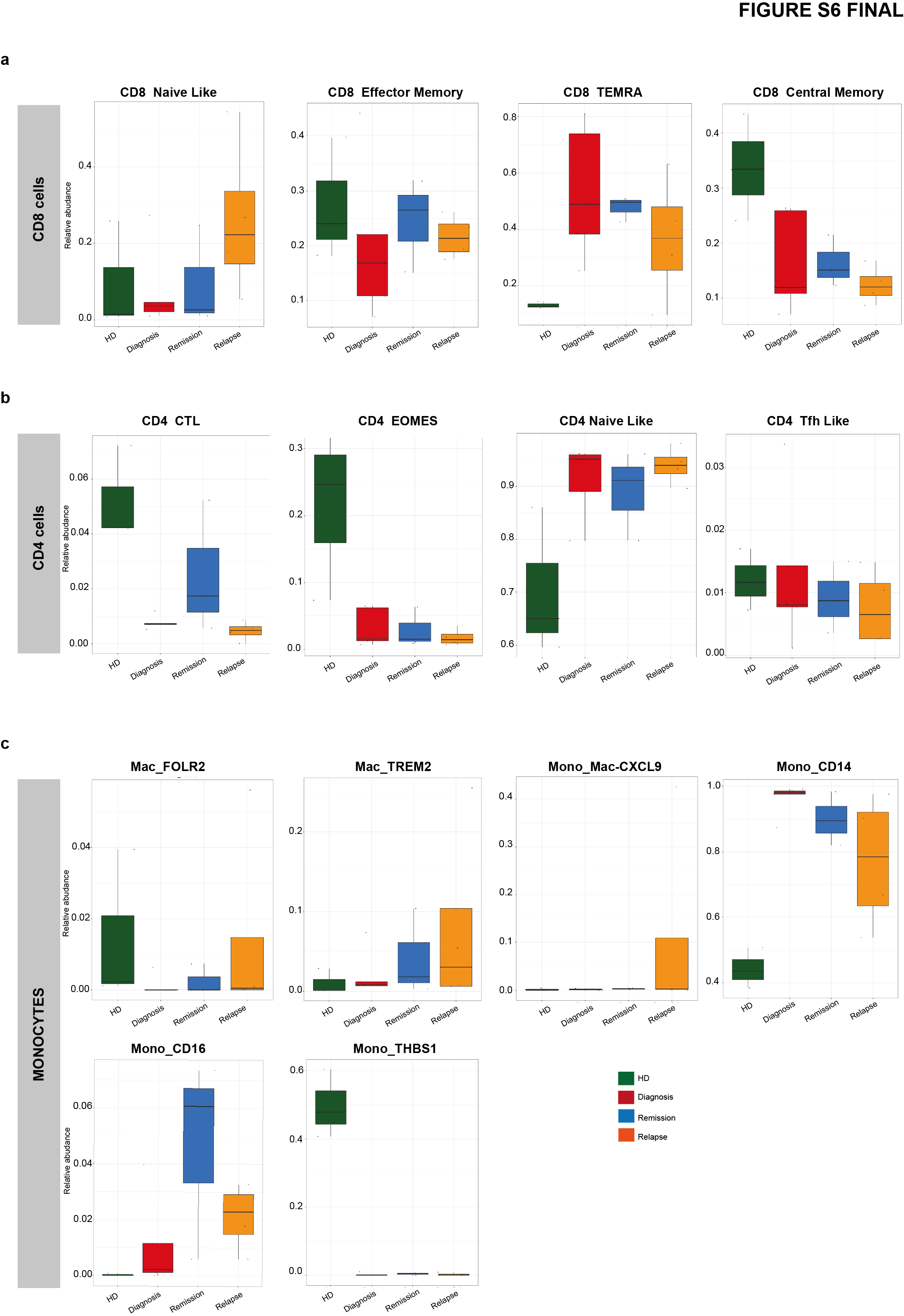


**Figure S6. Cell abundance in AHIA patients and Healthy Donors across clinical conditions**
(a-c) Box plots depicting the relative abundance of CD8 (a), CD4 (b), and monocyte (c) cells in AHIA patients and Healthy Donors (HDs) across clinical conditions: Green represents HDs, Red represents diagnosis, Blue represents remission, and Orange represents relapsed/refractory (RR).


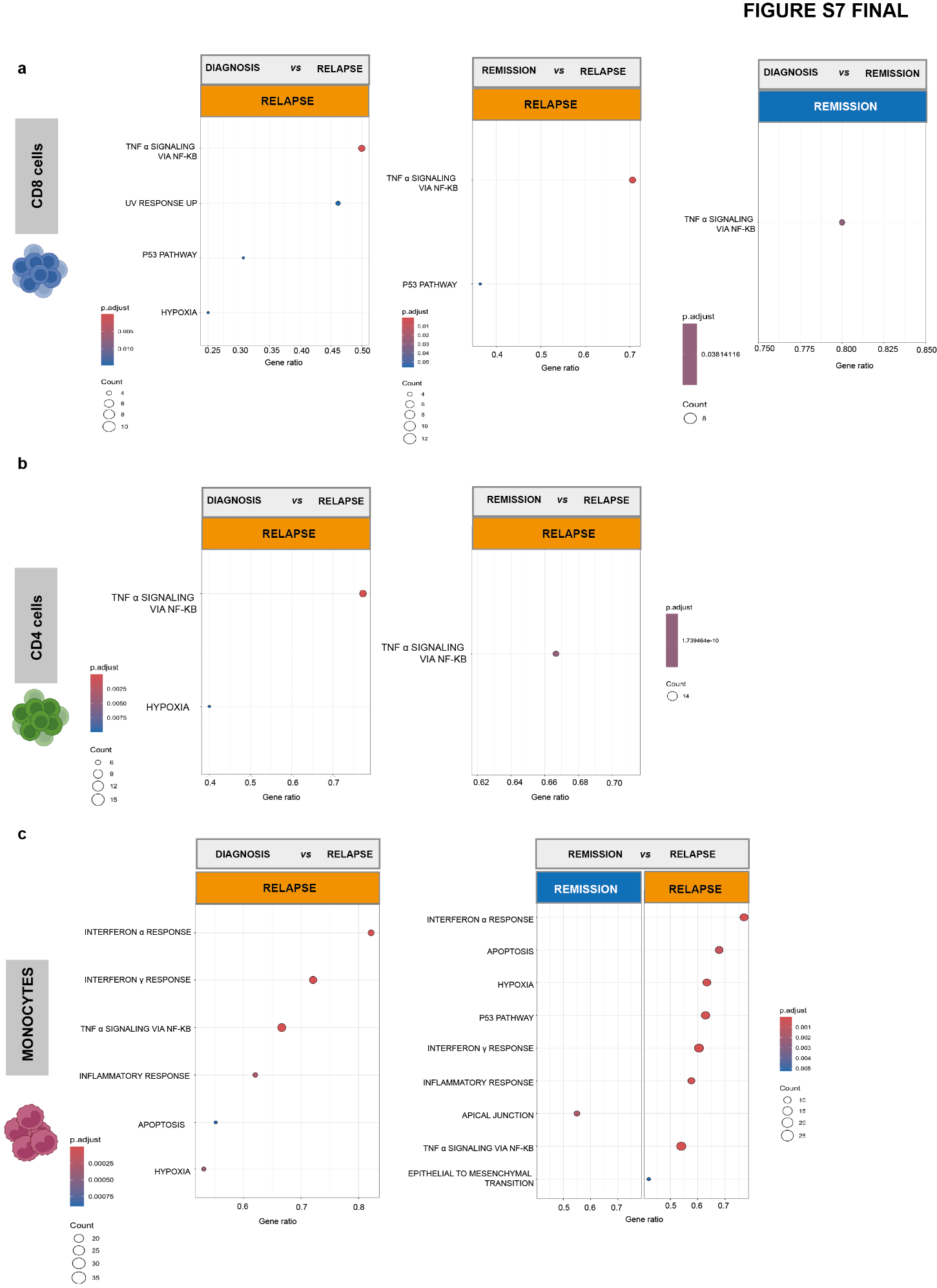


**Figure S7. Hallmark analysis of AHIA cell subpopulations**
(a-c) Differential expression analysis using the Wilcoxon rank-sum test to identify enriched Hallmark gene sets in T CD8+ cells (a), T CD4+ cells (b), and monocytes (c) across different clinical conditions. The y-axis indicates the gene ratio, dot size represents cell counts, and color gradient reflects the adjusted p-values (padj) in z-score.


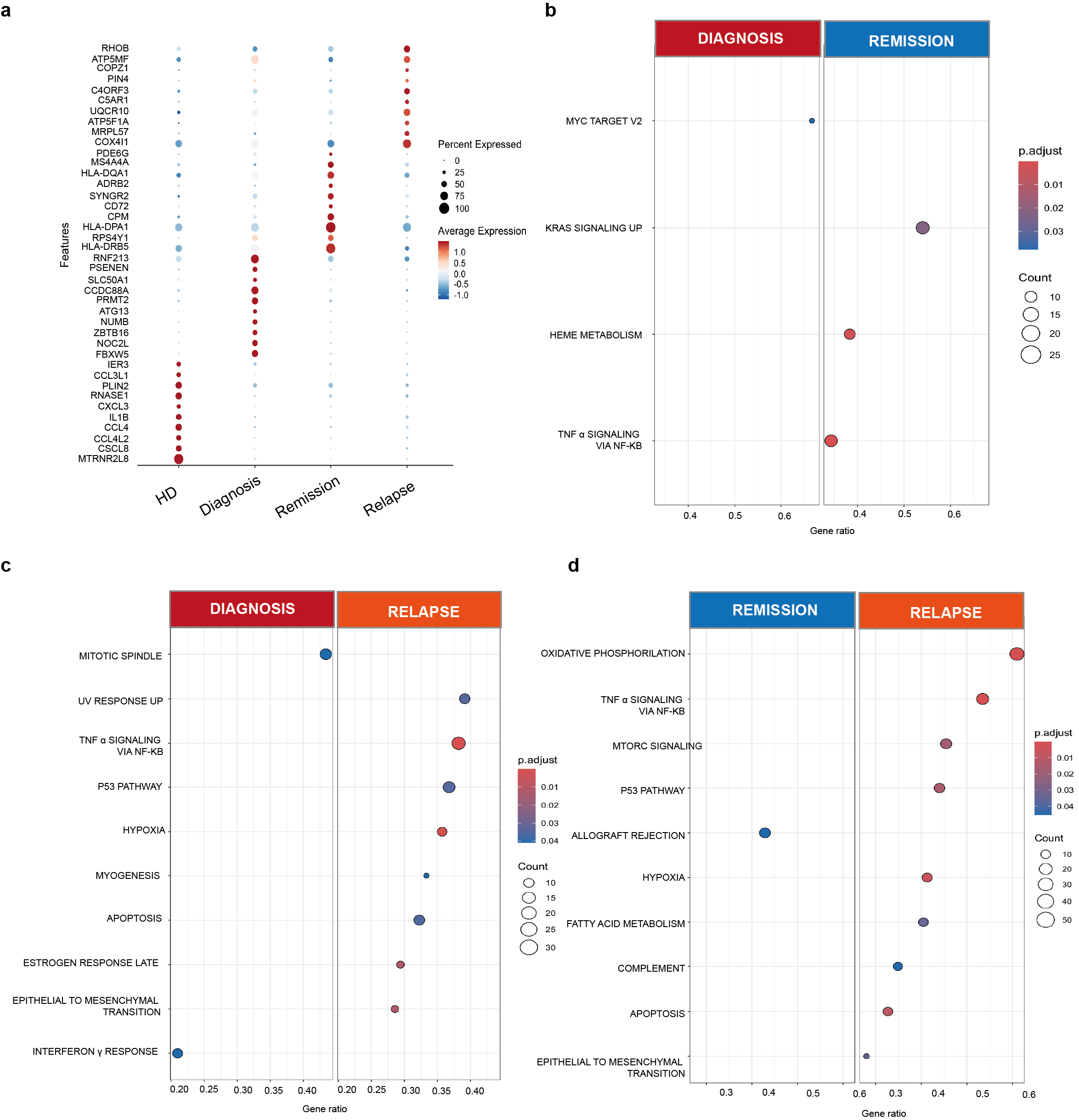


**Figure S8. Differential expression analysis of macrophages in AHIA patients across clinical status**
(a) Dot plot illustrating the top 15 marker genes that distinguish macrophage populations across clinical states. The x-axis represents the clinical categories, while the y-axis lists the gene names. Circle size indicates the number of cells expressing each gene, and the color intensity reflects the level of gene expression.
(b-d) Differential expression analysis using the Wilcoxon rank-sum test to identify enriched Hallmark gene sets across clinical groups: (b) diagnosis vs. remission, (c) diagnosis vs. relapsed/refractory (RR), and (d) remission vs. relapsed/refractory. The y-axis shows the gene ratio, with dot size representing the number of counts, and color indicating the adjusted p-values (padj) in z-score.


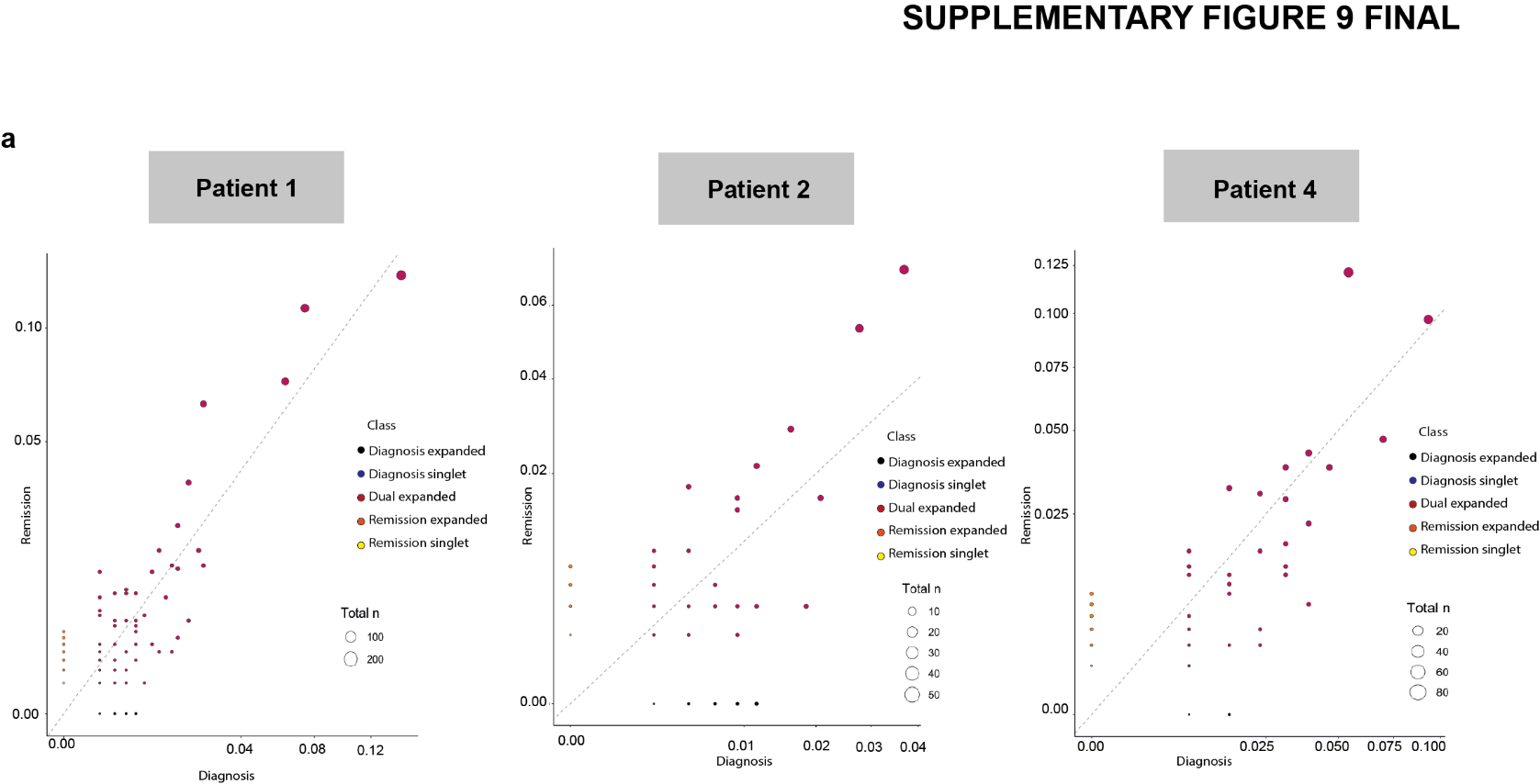


**Figure S9. Correlation of T CD8+ Clones at Diagnosis and Remission in AHIA Patients, Analyzed by Single-Cell RNA and TCR Sequencing (scRNA/TCR-seq)**


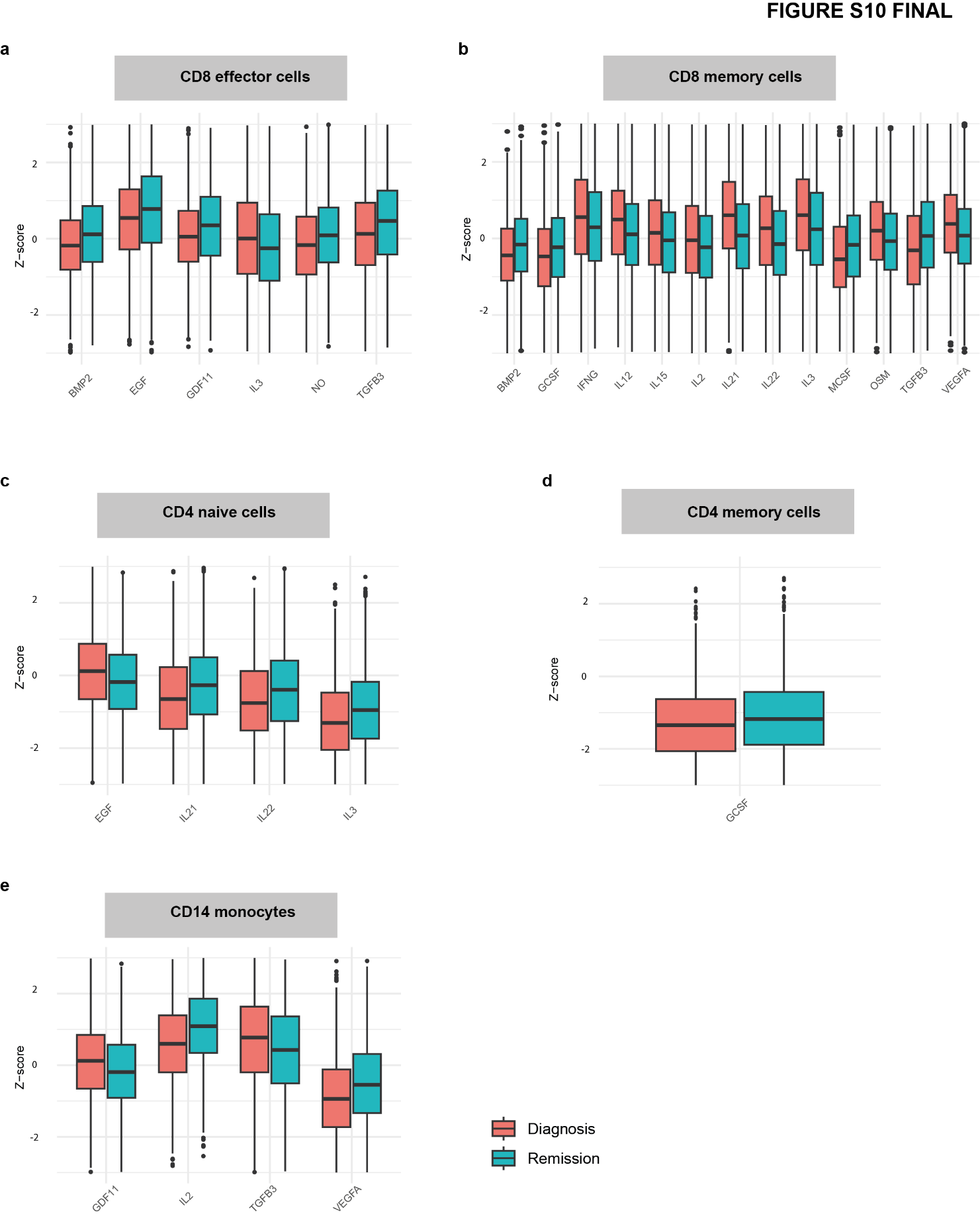


**Figure S10. Cytokine levels in different cellular subtypes at diagnosis versus remission**
Cytokine levels were measured across various cellular subtypes at diagnosis and remission timepoints. The reported cytokines show a statistically significant difference with a Z-score delta of at least 0.25 and a p-value <0.001, determined by the Tukey Honestly Significant Difference (HSD) test. These cytokines were selected for their relevance in distinguishing the clinical states.

Figure. S11.
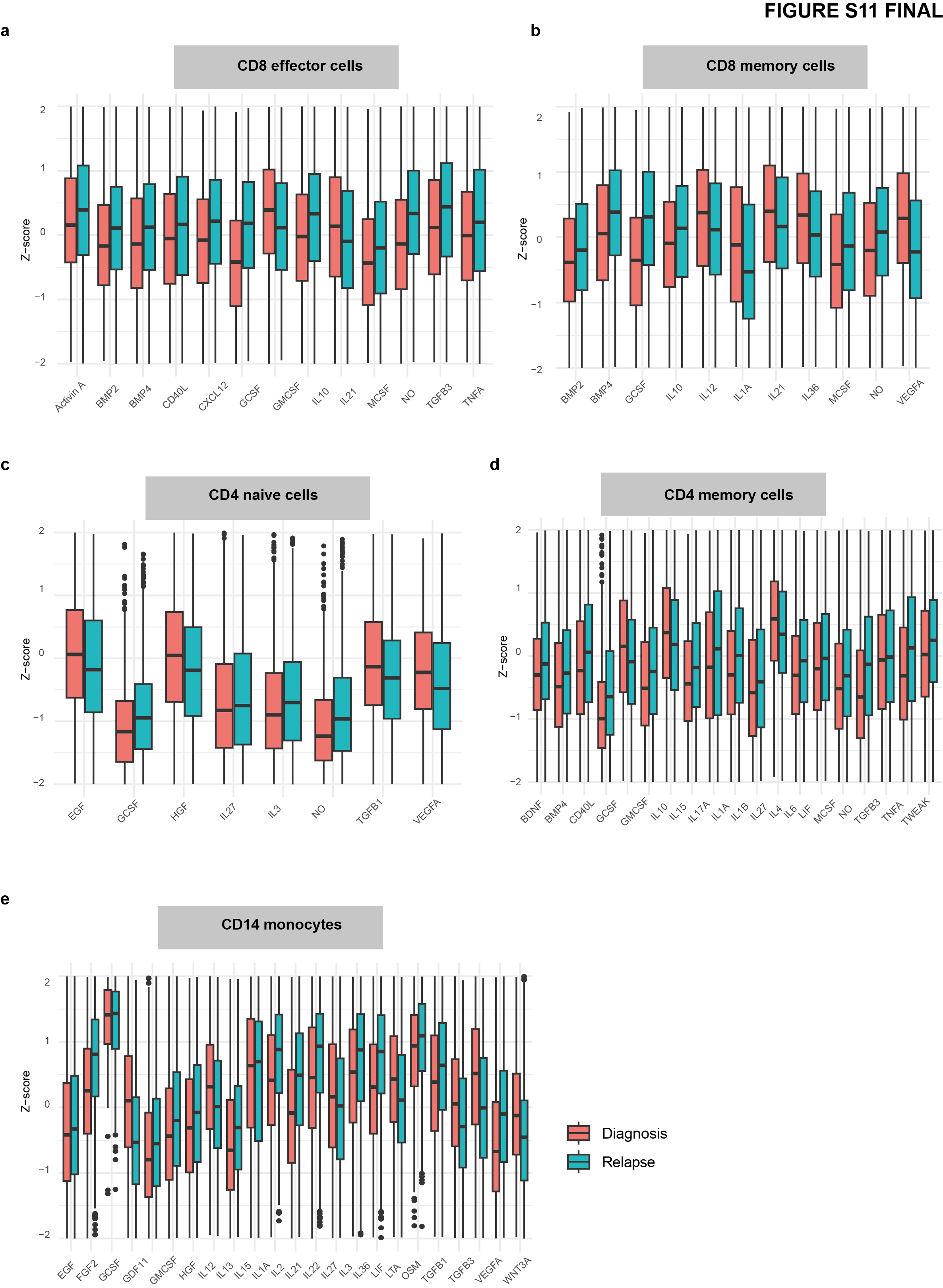


**Figure S11. Cytokine levels in different cellular subtypes at diagnosis versus relapse**
Cytokine levels were assessed in different cellular subtypes at diagnosis and relapse timepoints. Cytokines with a Z-score delta of at least 0.25 and a p-value <0.001, as determined by the Tukey Honestly Significant Difference (HSD) test, are reported here. These cytokines were selected for their statistically significant differences across timepoints and their potential role in disease progression.

| **Supplementary table 1. Demographic and hematologic characteristics of patients at enrollment according to AIHA type** | | | | | |
| --- | --- | --- | --- | --- | --- |
|  | **All**  **(N=97)** | **Warm AIHA (N=43)** | **Cold AIHA^ (N=44)** | **Mixed AIHA**  **(N=3)** | **Atypical AIHA^^ (N=7)** |
| **Median age, years (range)** | 59  (10-89) | 64  (10-85) | 58  (20-89) | 36  (58-30) | 57  (33-81) |
| **Male,n (%)** | 41  (42) | 23  (53) | 14  (32) | 1  (33) | 3  (43) |
| **Female n (%)** | 56  (58) | 20  (47) | 30  (68) | 2  (67) | 4  (57) |
| **Median follow-up, months (range)** | 42  (1-396) | 36  (1-396) | 46  (1-299) | 44  (31-53) | 27  (5-88) |
| **Hb (g/dL)** | 7.9  (3.0-11.6) | 7.1  (3.8-10.5) | 8.5  (3.0-11.6) | 8.3  (7.3-9.3) | 7.3  (4.0-9.7) |
| **LDH (U/L)** | 478  (155-2617) | 511  (182-2617) | 400  (155-1763) | 540  (224-1500) | 262  (172-382) |
| **Unconjugated bilirubin (mg/dL)** | 1.8  (0.3-9.6) | 1.8  (0.3-9.6) | 2.1  (0.4-6.7) | 2.5  (0.9-3.4) | 1.3  (0.9-2.5) |
| **Ret (x10^9/L)** | 189  (4-660) | 256.5  (15-623) | 150  (4.3-660) | 43  (23.9-140) | 220  (161-616) |
| **BMRI**** | 97  (4-427) | 121  (5-312) | 90  (4-325) | 29  (12-73) | 87  (76-427) |
| **Inadequate reticulocytes*****  **n (%)** | 36/64  (56) | 14/32  (44) | 16/25  (64) | 3/3  (100) | 3/4  (75) |
| **EPO (U/L)** | 54.7  (10-843) | 64.5  (16-843) | 52.1  (10-226) | 21.5  (21.5-21.5) | 37.7  (18.2-55.9) |
| **Inadequate EPO n (%)** | 47/63  (75) | 21/30  (70) | 22/28  (79) | 1/1  (100) | 3/4  (75) |

***BMRI<121; AIHA: autoimmune hemolytic anemia; Hb: hemoglobin; LDH: Lactate dehydrogenase; Hapto: haptoglobin; Ret: reticulocytes; EPO: endogenous erythropoietin;

* Values are expressed as median (range), unless otherwise specified

**BMRI = bone marrow responsiveness index = [absolute reticulocyte count x (patient’s Hb/normal Hb)]/1000

^5 patients had a condition associated with their cAIHA: 3 had associated chronic lymphocytic leukemia and 2 had a IgG monoclonal gammopathy of unknown significance.

^^6 patients had DAT negative AIHA, 1 had IgA positive AIHA.

| **Supplementary table 2. Therapies and related responses basing on bone marrow characteristics**  **Values expressed as n (%)** | | | | | | |
| --- | --- | --- | --- | --- | --- | --- |
|  | **Normo/**  **hypocellularity**  **(N=34)** | **Hypercellularity**  **(N=63)** | **Diserythropoiesis**  **(N=74)** | **No diserythropoiesis**  **(N=23)** | **MF=0**  **(N=69)** | **MF=1**  **(N=28)** |
| **First line** | | | | | | |
| **Transfusions** | 8 (24) | 29 (46) | 28 (38) | 9 (39) | 28 (41) | 98 (32) |
| ***Response*** | 8 (100) | 28 (97) | 27 (96) | 9 (100) | 27 (96) | 9 (100) |
| **IVIG** | 4 (12) | 8 (13) | 8 (11) | 4 (17) | 8 (12) | 4 (14) |
| ***Response*** | 4 (100) | 5 (63) | 6 (75) | 2 (50) | 6 (75) | 3 (75) |
| **Steroids** | 28(82) | 55 (87) | 66 (89) | 17 (74) | 59 (86) | 24 (86) |
| ***Response*** | 28 (100) | 43 (78) | 56 (85) | 15 (88) | 51 (86) | 20 (83) |
| **rEPO** | 2 (6) | 6 (10) | 7 (9) | 1 (4) | 6 (9) | 2 (7) |
| ***Response*** | 2 (100) | 5 (83) | 6 (86) | 1 (100) | 5 (83) | 2 (100) |
| **Rituximab** | 6(18) | 17(27) | 16 (22) | 7 (30) | 18 (26) | 5 (18) |
| ***Response*** | 6 (100) | 15 (88) | 14 (88) | 7 (100) | 16 (89) | 5 (100) |
| **Further lines** | | | | | | |
| **Rituximab** | 23 (68) | 43 (68) | 49 (66) | 17 (74) | 49 (71) | 17 (61) |
| ***Response*** | 23 (100) | 39 (91) | 45 (92) | 17 (100) | 46 (94) | 16 (94) |
| **Immunosuppressors** | 9 (26) | 16 (25) | 20 (27) | 5 (22) | 20 (29) | 5 (18) |
| ***Response*** | 7 (78) | 8 (50) | 12 (60) | 3 (60) | 14 (70) | 1 (20) |
| **Splenectomy** | 0 (0) | 3 (5) | 3 (4) | 0 (0) | 1 (1) | 2 (7) |
| ***Response*** |  | 3 (100) | 3 (100) |  | 1 (100) | 2 (100) |
| **rEPO** | 9 (26) | 18 (29) | 21 (28) | 6 (26) | 18 (26) | 9 (32) |
| ***Response*** | 6 (67) | 15 (83) | 16 (76) | 5 (83) | 15 (83) | 6 (67) |
| MF0: without marrow fibrosis; MF1: reticulin fibrosis; CR: complete response (Hb>12g/dl); PR: partial response (Hb>10g/dl or Hb increase >2g/dl); IVIG: intravenous immunoglobulin; AZA: azathioprine; CP: cyclophosphamide; CyA: cyclosporine; rEPO: recombinant erythropoietin | | | | | | |

| **Supplementary table 3. Therapies and related responses basing on the lymphoid infiltrate.**  **Values expressed as n (%)** | | | | |
| --- | --- | --- | --- | --- |
|  | **B-cells**  **(N=8)** | **T-cells**  **(N=40)** | **Mixed**  **(N=34)** | **No lymphoid infiltrate**  **(N=15)** |
| **First line** | | | | |
| **Transfusions** | 3 (38) | 18 (45) | 12 (35) | 4 (27) |
| ***Response*** | 3 (100) | 17 (94) | 12 (100) | 4 (100) |
| **IVIG** | 0 (0) | 4 (10) | 5 (15) | 3 (20) |
| ***Response*** |  | 3 (75) | 4 (80) | 2 (67) |
| **Steroids** | 6 (75) | 36 (90) | 27 (79) | 14 (93) |
| ***Response*** | 4 (67) | 29 (81) | 25 (93) | 13 (93) |
| **rEPO** | 0 (0) | 6 (15) | 1 (3) | 1 (7) |
| ***Response*** |  | 5 (83) | 1 (100) | 1 (100) |
| **Rituximab** | 4 (50) | 9 (23) | 8 (24) | 2 (13) |
| ***Response*** | 4 (100) | 7 (78) | 8 (100) | 2 (100) |
| **Further lines** | | | | |
| **Rituximab** | 6 (75) | 27 (68) | 24 (71) | 9 (60) |
| ***Response*** | 6 (100) | 24 (89) | 23 (96) | 9 (100) |
| **Immunosuppressors** | 2 (25) | 12 (30) | 8 (24) | 3 (20) |
| ***Response*** | 1 (50) | 7 (58) | 6 (75) | 1 (33) |
| **Splenectomy** | 0 (0) | 3 (8) | 0 (0) | 0 (0) |
| ***Response*** |  | 3 (100) |  |  |
| **rEPO** | 3 (38) | 12 (30) | 7 (21) | 5 (33) |
| ***Response*** | 2 (67) | 9 (75) | 6 (86) | 4 (80) |
| Values expressed as n (%); CR: complete response (Hb>12g/dl); PR: partial response (Hb>10g/dl or Hb increase >2g/dl); IVIG: intravenous immunoglobulin; AZA: azathioprine; CP: cyclophosphamide; CyA: cyclosporine; rEPO: recombinant erythropoietin | | | | |
